# LeishIF4E-5 Is a Promastigote-Specific Cap-Binding Protein in *Leishmania*

**DOI:** 10.3390/ijms22083979

**Published:** 2021-04-12

**Authors:** Rohit Shrivastava, Nitin Tupperwar, Bar Schwartz, Nofar Baron, Michal Shapira

**Affiliations:** 1 Department of Life Sciences, Ben-Gurion University of the Negev, Beer-Sheva 84105, Israel; biorohit@gmail.com (R.S.); nitinvet@gmail.com (N.T.); barschw@post.bgu.ac.il (B.S.); baronn@post.bgu.ac.il (N.B.); 2 CSIR-Centre for Cellular and Molecular Biology, Hyderabad 50007, India

**Keywords:** *Leishmania*, protein synthesis, translation regulation, LeishIF4E-5, LeishIF4G

## Abstract

*Leishmania* parasites cycle between sand fly vectors and mammalian hosts, transforming from extracellular promastigotes that reside in the vectors’ alimentary canal to obligatory intracellular non-motile amastigotes that are harbored by macrophages of the mammalian hosts. The transition between vector and host exposes them to a broad range of environmental conditions that induces a developmental program of gene expression, with translation regulation playing a key role. The *Leishmania* genome encodes six paralogs of the cap-binding protein eIF4E. All six isoforms show a relatively low degree of conservation with eIF4Es of other eukaryotes, as well as among themselves. This variability could suggest that they have been assigned discrete roles that could contribute to their survival under the changing environmental conditions. Here, we describe LeishIF4E-5, a LeishIF4E paralog. Despite the low sequence conservation observed between LeishIF4E-5 and other LeishIF4Es, the three aromatic residues in its cap-binding pocket are conserved, in accordance with its cap-binding activity. However, the cap-binding activity of LeishIF4E-5 is restricted to the promastigote life form and not observed in amastigotes. The overexpression of LeishIF4E-5 shows a decline in cell proliferation and an overall reduction in global translation. Immuno-cytochemical analysis shows that LeishIF4E-5 is localized in the cytoplasm, with a non-uniform distribution. Mass spectrometry analysis of proteins that co-purify with LeishIF4E-5 highlighted proteins involved in RNA metabolism, along with two LeishIF4G paralogs, LeishIF4G-1 and LeishIF4G-2. These vary in their conserved eIF4E binding motif, possibly suggesting that they can form different complexes.

## 1. Introduction

*Leishmania* parasites cycle between sand fly vectors and mammalian hosts, transforming from extracellular flagellated and motile promastigotes in the female sand flies to obligatory intracellular non-motile amastigotes that reside within macrophages of the mammalian hosts. The transition between vector and host exposes the parasites to a broad range of environmental conditions that induces a developmental program of gene expression, with translation regulation playing a key role. The alternation between the sandfly vector and mammalian host is as an integral part of their life cycle. These transitions between two different hosts are accompanied by shifts in environmental conditions, mainly, extreme changes in temperature and pH [1]. In higher eukaryotes, such changes usually cause a cellular stress that may lead to protein misfolding and aggregation. To prevent these problems, cells employ a global arrest of cap-dependent translation that stops the de novo synthesis of most proteins, except for proteins that are essential for overcoming the encountered stress. These continue to be translated in a cap-independent manner [2,3,4,5].

In eukaryotes, translation initiation is a highly complex and regulated process that involves multiple initiation factors, including the recruitment of an eIF4F complex to the 5′ cap structure of mRNA. The eIF4F complex consists of eIF4E, a cap-binding protein; eIF4G, a scaffold protein that serves as a hub for eIF4E; eIF4A, an RNA helicase that unwinds the mRNA secondary structure; eIF3 that recruits the 40S ribosomal subunit, and the poly (A)-binding protein (PABP) [6].

The *Leishmania* genome encodes six different paralogs of the cap-binding protein eIF4E and five paralogs of its eIF4G-binding partners. The six different isoforms of the *Leishmania* eIF4E show a relatively low degree of conservation with eIF4E of other eukaryotes, or among themselves [7]. The low conservation observed between the different paralogs could suggest that each is involved in distinct functions that drive differential gene expression, enabling survival in the vector and host. Although the functions of LeishIF4Es 1–4 have been investigated, some overlaps in their functions are expected. LeishIF4E-4 is considered to be a canonical initiation factor as it binds efficiently to the cap-4 structure, which is the unique mRNA cap in trypanosomatids [8,9], and associates with LeishIF4G-3, one of the five LeishIF4G candidates [10]. However, under conditions that induce differentiation to axenic amastigotes, LeishIF4E-4 loses its cap-binding activity [11]. Another cap-binding factor, LeishIF4E-1, also binds cap-4 efficiently [12], but it does not associate with any of the LeishIF4G candidates. Alternatively, it binds a unique Leish4E-IP1 regulatory protein that inhibits its ability to bind the cap-structure [11,13]. LeishIF4E-1 expression increases at elevated temperatures [7] and it is the only LeishIF4E paralog that continues to bind m^7^GTP following differentiation to axenic amastigotes. Recently, we have reported that LeishIF4E-1 is a potent translation initiation factor in the promastigote stage of *Leishmania* parasites and that its deletion by CRISPR-Cas9 led to a decrease in the promastigote specific proteome [14].

LeishIF4E-3 is another cap-binding paralog, and it interacts with LeishIF4G-4 under normal growth conditions. However, during nutritional stress the two proteins dissociate, and LeishIF4E-3 enters into cytoplasmic granules that store inactive ribosomal subunits and mRNAs [15,16]. The deletion of a single allele of LeishIF4E-3 affected global translation and parasite infectivity in a cultured macrophage cell line, suggesting that LeishIF4E-3 has a role in translation under normal conditions [17].

LeishIF4E-2 is one of the less-studied paralogs of eIF4E. It does not appear to bind any of the eIF4G candidates and was found to migrate with the heavy polysomal complexes in sucrose gradients, thus could be associated with the stabilization of polysomes in *Leishmania*. Genome-wide tethering assay in *Trypanosoma brucei* classified LeishIF4E-2 as a putative translation repressor [18], but in *Leishmania* its role still remains elusive [19].

Two additional isoforms of eIF4E have been identified in *Trypanosoma (T.) brucei*, TbIF4E-5 and TbIF4E-6 [20,21], and their *Leishmania* counterparts, LeishIF4E-5 and LeishIF4E-6, were also identified. In *T. brucei*, EIF4E-6 forms a complex with the eIF4G homolog, TbIF4G-5, and with a 70.3 kDa hypothetical protein referred to as TbG5-IP. TbIF4E-5 associates with TbIF4G-1 and TbIF4G-2, forming two different complexes. However, the role of these complexes has not yet been resolved. The crystal structure of the *T. cruzi* TcIF4E-5 in complex with cap-4 has been solved, highlighting the relevant interactions that stabilize its cap-binding activity [22].

Here, we investigate LeishIF4E-5, the *Leishmania* ortholog of TbIF4E-5. We show that although it binds the m^7^GTP cap analog, this binding is restricted to the promastigote life form. The overexpression of LeishIF4E-5 in transgenic parasites reduces global translation and cell proliferation, excluding the possibility that LeishIF4E-5 is a canonical translation factor. Pull-down experiments highlighted its interacting partners, among them LeishIF4G-1 and LeishIF4G-2, as also observed for TbIF4E-5, the trypanosome ortholog. These experiments also highlight that LeishIF4E-5 associates with proteins related to RNA metabolism as well as with other LeishIF4Es.

## 2. Results

### 2.1. Sequence Conservation of LeishIF4E-5

**Figure 1:**
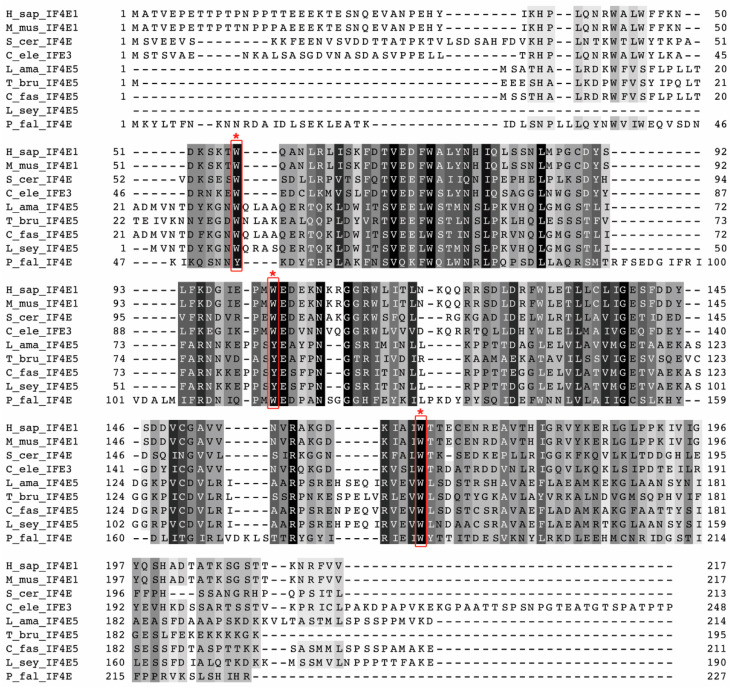
**Alignment of LeishIF4E-5 from *L. amazonensis* with its protozoan orthologs, with eIF4E-1 from mammals, with yeast eIF4E and *C. elegans* IFE-3**. The alignment includes sequences of LeishIF4E-5 from *Leishmania amazonensis* (L_ama_IF4E5)*, Trypanosoma brucei* (T_bru_IF4E5), *Crithidia fasciculata* (C_fas_IF4E5) and *Leptomonas seymouri* (L_sey_IF4E5). It also includes the sequences of the canonical eIF4E from *Homo sapiens* (H_sap_IF4E-1), *Mus musculus* (M_mus_IF4E-1) and *Saccharomyces cerevisiae* (S_cer_IF4E5) along with IFE-3 from *C. elegans* (C_ele_IFE3) and *P. falciparum* (P_fal_IF4E). All sequences were subjected to multiple sequence alignment to highlight their homologies. Conserved tryptophan residues involved in cap-binding are marked with asterisks. The multiple sequence alignment was performed using MAFFT, version 7. Sequence conservations were generated by Jalview and are highlighted in greyscale.

The *Leishmania* ortholog of the trypanosomatid TbIF4E-5 [21], LeishIF4E-5, was identified in the genome of *L. amazonensis*. Figure 1 shows the alignment of LeishIF4E-5 from *L. amazonensis* with TbIF4E-5 of *T. brucei* and eIF4E-1 of *Mus musculus* and other eIF4E orthologs. The cap-binding pocket of the mouse eIF4E contains three conserved aromatic residues, with three tryptophans occupying positions 56, 102 and 166 [23]. These are also found in the single yeast eIF4E, in the *Plasmodium falciparum* protein and in the canonical eIF4E of *C. elegans*, denoted IFE-3. The *L. amazonensis* LeishIF4E-5 and its orthologs from *T. brucei* and from different non-pathogenic kinetoplastids (*Leptomonas seymouri and Crithidia fasciulata*) have aromatic residues at parallel positions: in *L. amazonensis*, the Trp residues are found at positions 32 and 151, and a Tyr is at position 83. In *T. brucei*, the two Trp residues are at positions 33 and 151 and the Tyr residue is at position 83. In all the kinetoplastid orthologs of LeishIF4E-5 examined, the residue that occupies the position parallel to the human Trp102 is Tyr. The aromatic residues in these three conserved positions of the cap-binding pocket are considered responsible for the stacking interactions with the m^7^G. We further compared between LeishIF4E-5 and other LeishIF4Es as well as with the mouse eIF4E-1 and observed a high similarity between LeishIF4E-5 and LeishIF4E-6 (43.2%) and a lower similarity to other eIF4Es in *Leishmania* and mouse (Figure S1).

The comparison between the different proteins is also reflected in the phylogenetic tree (Figure S2). The tree emphasizes the evolutionary relationship between the different kinetoplastid orthologs of LeishIF4E-5 that are clustered together, whereas the mammalian eIF4E-1 appears to cluster with the *C. elegans* canonical IFE-3. The eIF4E from yeast and *P. falciparum* appear to be more distant, but closer to the mammalian proteins than to the kinetoplastid eIF4E-5, possibly since these are canonical translation factors. Additionally, *P. falciparum* belongs to the phylum Apicomplexa, a distinct taxonomic group of single celled parasites, within the order Haemosporida. *Leishmania*, however, belong to the order Kinetoplastida, which includes pathogenic and non-pathogenic organisms, all containing the unique kinetoplast organelle carrying the mitochondrial genome [24].

### 2.2. Episomal Expression of LeishIF4E-5 Affects Cellular Growth and Global Translation

**Figure 2:**
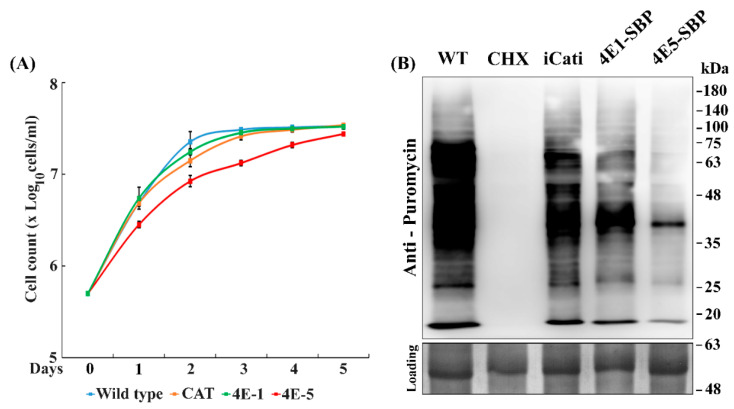
**Episomal expression of LeishIF4E-5 affects global translation and growth.** Wild type *L. amazonensis,* transgenic *L. amazonensis* promastigotes expressing chloramphenicol acetyltransferase (CAT), SBP-tagged LeishIF4E-5 and SBP-tagged LeishIF4E-1 were grown under normal conditions. (**A**) Cell counts were taken during 5 days. Cells expressing LeishIF4E-5 tagged with the streptavidin binding peptide, SBP (4E5-SBP), are shown in red, LeishIF4E-1-SBP cells are shown in green, cells expressing the CAT reporter are shown in orange and wild type cells are shown in blue. Cell growth is represented by log_10_ cells/mL against the number of days, taken in triplicates. Lines were drawn based on the mean values of the different experiments with standard errors. (**B**) Cells were incubated with 1 µg/mL puromycin for 20 min, extracted proteins were separated over 12% SDS-PAGE and subjected to Western analysis using specific antibodies against puromycin. A cycloheximide (CHX) control for complete translation inhibition is also shown. The different lanes contain equal protein loads, as shown by the Ponceau staining (bottom panel). The experiment was repeated three times and the densitometric analysis is given in Figure S3.

To monitor the effects of LeishIF4E-5 episomal expression on *Leishmania* growth, we measured the proliferation of stably transfected cells that expressed the Streptavidin binding peptide (SBP)-tagged LeishIF4E-5. Control lines expressed the chloramphenicol acetyltransferase (CAT) reporter protein, the SBP-tagged LeishIF4E-1, and wild type (WT) parasites. Figure 2A shows that cell lines expressing the SBP-tagged LeishIF4E-5 had a slight growth decay, while growth of the control SBP-tagged LeishIF4E-1 cells or cells expressing the CAT reporter resembled that of WT cells.

To investigate whether the expression of the SBP-LeishIF4E-5 affected overall translation, we used the SUnSET assay, which is based on the incorporation of puromycin into growing polypeptide chains. The integration of puromycin into growing polypeptide chains was monitored using specific antibodies against puromycin in Western blots that were loaded with equal amounts of proteins. As observed in Figure 2B, expression of the transgenic LeishIF4E-5 led to a reduction in global translation when compared to control lines that expressed the CAT reporter and the SBP-LeishIF4E-1. The comparison of all lines to the WT control cells showed that the episomal overexpression of different transgenes led to a reduction in global translation. However, a much stronger effect was observed with cells that expressed SBP-LeishIF4E-5, as compared to the control lines that overexpressed transgenic SBP-LeishIF4E-1, or the non-related CAT reporter gene (Figure S3). The overexpression of transgenes may often have a profound effect on global translation [14,17], possibly due to competition over components of the translation machinery. However, the densitometric analysis indicated a stronger effect of SBP-LeishIF4E-5 expression on global translation as compared with WT (67.6% inhibition). The inhibition of global translation in the other transgenic control cell lines was 25.7% for LeishIF4E-1 and 18.4% for cells that expressed the CAT reporter (Figure S3).

### 2.3. LeishIF4E-5 Shows a Non-Uniform Distribution in the Cytoplasm

**Figure 3:**
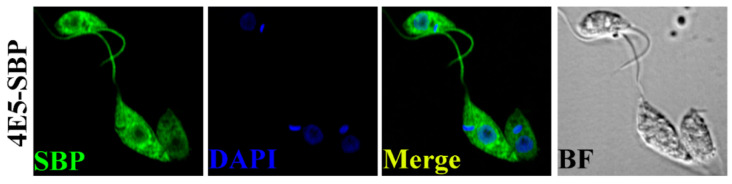
**Non-uniform distribution of SBP-tagged LeishIF4E-5 in the cytoplasm and in foci surrounding the nucleus.** Transgenic *L. amazonensis* promastigotes expressing SBP-tagged LeishIF4E-5 were fixed, permeabilized and processed for confocal microscopy. LeishIF4E-5-SBP was detected using a monoclonal antibody against the SBP tag and a secondary DyLight-labeled antibody (488 nm; green). Nuclear and kinetoplast DNA was stained using DAPI (blue). A bright field (BF) picture of the cells is on the right. The confocal analysis was repeated three times. A broad view of the field is given in Figure S4.

The sub-cellular localization of the protein can provide a general understanding of its function. We monitored the localization of SBP-tagged LeishIF4E-5 in transgenic parasites by confocal analysis using antibodies against the SBP tag. Figure 3 and Figure S4 show that LeishIF4E-5 was localized in the cytoplasm in a non-uniform distribution pattern that highlighted its concentration in cytoplasmic foci; several foci were also observed around the nucleus. Such distribution could indicate that LeishIF4E-5 is involved in quality control of mRNAs that are exported from the nucleus.

### 2.4. The Cap-Binding Activity of LeishIF4E-5 Is Restricted to the Promastigote Life Stage

**Figure 4:**
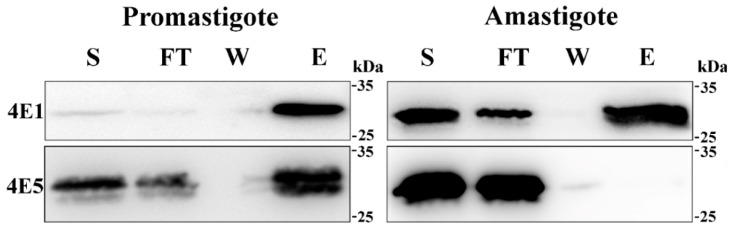
**LeishIF4E-5 binds to the cap analog m^7^GTP only during the promastigote stage.** Transgenic *L. amazonensis* promastigotes expressing SBP-tagged LeishIF4E-5 were lysed and incubated with m^7^GTP-agarose beads. The beads were washed and eluted using 200 μM free m^7^GTP. Samples from the eluted protein fractions and washes were precipitated by 10% trichloro-acetic acid (TCA). The gels were loaded with samples of the total supernatant (S, 2%), flow-through (FT, 2%), the wash (W, 50%) and the eluted fractions (E, 50%). The proteins were resolved over 12% SDS-PAGE and analyzed using specific antibodies against the SBP-tag to identify LeishIF4E-5 and antibodies raised against LeishIF4E-1. Similar results were obtained from three independent experiments. Densitometric analysis of the cap-binding activity in promastigotes is given in Figure S5.

To further understand the potential role of LeishIF4E-5 during translation, its m^7^GTP-binding activity was monitored in promastigotes and axenic amastigotes. Extracts of cells expressing SBP-tagged LeishIF4E-5 and SBP-tagged LeishIF4E-1 for control were obtained from mid-log promastigotes and axenic amastigotes. The extracts were affinity-purified over m^7^GTP-agarose beads that were further eluted with free m^7^GTP. Aliquots from the supernatant, flow-through, wash, and the eluted fractions were subjected to Western analysis using specific antibodies against the SBP-tag. LeishIF4E-1 served as a positive control for binding to m^7^GTP at both life forms as previously reported [11]. Figure 4 shows that the cap-binding activity of LeishIF4E-5 was observed only in promastigotes and not in axenic amastigotes, whereas the binding of the LeishIF4E-1 control to m^7^GTP was retained in both life forms. The blots were subjected to densitometric analysis and the cap-binding activity was evaluated first by calculating the ratios between the eluted (E) and supernatant (S) fractions and then by comparing the E/S ratios between cells that expressed LeishIF4E-5 with the E/S values of cells that expressed LeishIF4E-1, which served as a 100% control. The E/S value of LeishIF4E-5 was therefore calculated to be 9.5% as compared to the E/S ratio of cells expressing LeishIF4E-1 (Figure S5).

### 2.5. Identification of the LeishIF4E-5 Interactome 

**Figure 5:**
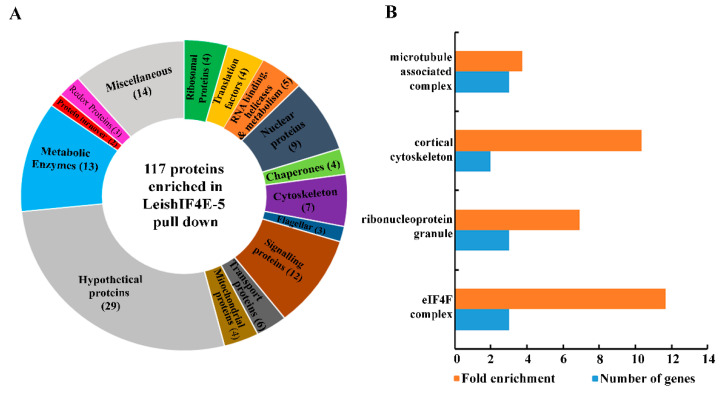
**Categorized proteins found enriched with LeishIF4E-5.** SBP-LeishIF4E-5 and its associated proteins were pulled-down over streptavidin-Sepharose beads. Control pull downs were performed with cells expressing SBP-luciferase. The proteomic content of the pulled-down extracts was assessed by LC-MS/MS. Proteins were identified using the MaxQuant software and their enrichment as compared to the luciferase control was determined using the Perseus statistical tool, highlighting a log_2_ fold change of ≥1.6, with an adjusted *p* value (Padj) < 0.05. A total of 117 proteins were found to be significantly enriched in the interactome of SBP-LeishIF4E-5 that served as the bait protein. The proteins were categorized into groups according to their annotated functions. (**A**) The pie chart shows the relative distribution of proteins that were manually categorized into functional groups. The pie chart shows the relative abundance of each category, based on the summed intensities of the peptides that were used to identify the individual proteins. The numbers in brackets in the pie chart represent the number of proteins in each category. (**B**) Gene Ontology (GO) term enrichment by cellular components. The resulting GO terms were enriched by at least three-fold as compared to the gene sets encoded in the genome, with a *p* value ≤ 0.05.

We examined the LeishIF4E-5 interacting proteins by pull-down experiments followed by mass spectrometry analysis. The presence of LeishIF4E-5 in the eluted fraction was verified by Western analysis using antibodies against the SBP-tag. The peptides generated from the pull-down of the SBP-tagged LeishIF4E-5 were identified based on the annotated *L. major* proteins in TriTrypDB. Control pull-downs were performed on cells expressing the SBP-tagged luciferase. The identified proteins were subjected to statistical analysis using Perseus software and proteins with a Log_2_ fold enrichment of 1.6 (three-fold change) and a *p* value < 0.05 were selected. The highlighted proteins were manually clustered into functional groups as shown in Figure 5A and Table S1. The proteins were further classified by their Gene Ontology (GO) enrichment, based on their cellular components as shown in Figure 5B and Supplement Table S2.

The LeishIF4E-5 interactome included cytoskeletal, signaling and nuclear proteins, along with several ribosomal proteins, translation factors, RNA helicases and RNA binding proteins. The manual classification also highlighted a relatively large number of metabolic proteins and proteins without any known function (hypothetical proteins, Figure 5A and Table S1). The GO term enrichment analysis also highlighted cytoskeletal proteins, proteins associated with cytoplasmic ribonucleoprotein granules and translation factors that are part of the eIF4F complex (Figure 5B and Table S2). Both analyses highlighted the enrichment of cytoskeletal and flagellar proteins, suggesting that LeishIF4E-5 could have a role in cell motility. The enrichment of numerous metabolic enzymes in the LeishIF4E-5 interactome is rather intriguing, but studies suggest that some metabolic enzymes could bind RNA even in the absence of canonical RNA binding motifs [25].

The LeishIF4E-5 interactome contained ATP-dependent RNA helicases and RNA binding proteins, and these proteins could be involved in the remodeling of ribonucleoprotein complexes. The LeishIF4E-5 interactome also contained proteins that have a function in RNA turn over. One is the exosome subunit rrp6 (LmjF.34.3080), this subunit is involved in RNA quality control (Table S1). The other is LmjF.22.1600, annotated by TriTrypDB as the *Leishmania* ortholog of the *T. brucei* decapping enzyme ApaH-like Phosphatase (TbALPH1, Tb927.6.640, [26]). Figure S6 shows the alignment of LmjF.22.1600 with TbALPH1 and with the *E. coli* ApaH, NP_414591.1, highlighting a sequence similarity of 44% between TbALPH1 and the *Leishmania* LmjF.22.1600 in a region that encompasses sequences between positions 340 and 488 of LmjF.22.1600. This region also contains three signature motifs (GDXHG, GDXXnRG and GNH(E/D)) [27] of protein phosphatases.

We noted the presence of several nuclear and transport proteins in the LeishIF4E-5 interactome, in accordance with the localization of this protein in foci that surround the nucleus. The presence of chaperones in the LeishIF4E-5 interactome is not surprising as chaperones usually accompany overexpressed proteins.

The mass spectrometry analysis of proteins that coeluted with LeishIF4E-5 revealed two LeishIF4G paralogs, LeishIF4G-1 and LeishIF4G-2 (Table S1). A comparison between the parasite LeishIF4Gs showed a high sequence similarity between LeishIF4G-1 and LeishIF4G-5 (30.4%, Figure S8A), whereas LeishIF4G-2 showed the highest similarity with the mouse eIF4G-1 (26.1%, Figure S8B). A domain search in LeishIF4G-1 and LeishIF4G-2 for the eIF4E interacting motif Y(X)_4_Lɸ (where ɸ is any hydrophobic residue), identified a putative motif only in LeishIF4G-2 but not in LeishIF4G-1. The eIF4E-interacting motif in LeishIF4G-2 was located towards the C-terminus of the protein (Figure S7A), whereas in LeishIF4G-3 it is located in the N-terminus [10]. We also noted that both TbIF4G-1 and TbIF4G-2 orthologs from *T. brucei* did contain the putative YXXXXLɸ 4E-binding motif, unlike LeishIF4G-1 (Figure S7B). TbIF4G-2 contains two putative 4E-binding motifs which bear no resemblance to the LeishIF4G-2 motifs. Two such motifs were also found in TbIF4G-3 but only one, whichcorresponds to positions 21–27, showed a complete sequence similarity to the element that we previously characterized in LeishIF4G-3 [10].

### 2.6. LeishIF4E-5 Interacts Directly with LeishIF4G-1 and LeishIF4G-2

**Figure 6:**
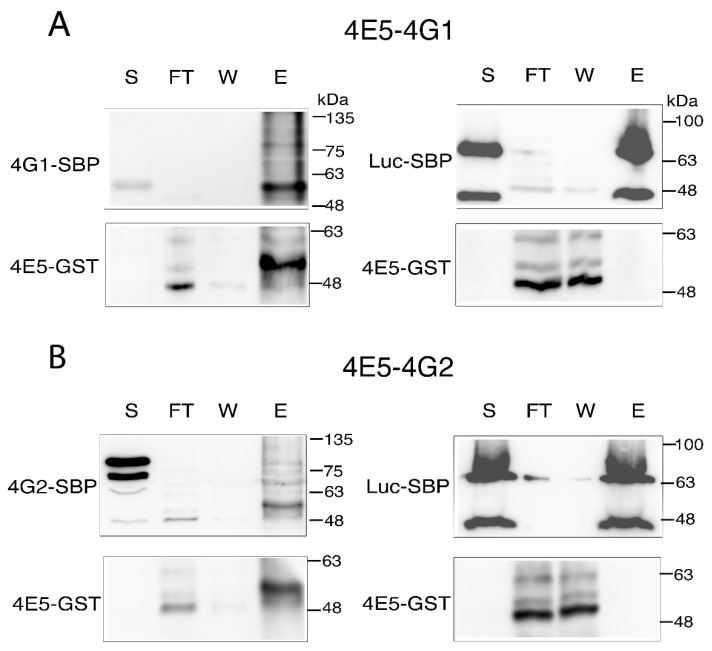
**Recombinant LeishIF4E-5-GST interacts directly with LeishIF4G-1 and LeishIF4G-2.** Lysates of *Leishmania* cell lines expressing SBP-tagged LeishIF4G-1 or LeishIF4G-2 were bound to Streptavidin beads, followed by incubation with *E. coli* cell lysates expressing recombinant GST-tagged LeishIF4E-5. After several washes, the final elution was carried out with 5 mM Biotin. *Leishmania* cell line expressing SBP-tagged luciferase was used as a negative control. Aliquots obtained from the supernatant (S, 2%), the flow-through (FT, 2%), the wash (W, 25%) and the eluted fractions (E, 25%) were resolved over 12.5% SDS-PAGE, and subjected to Western blot analysis with specific monoclonal antibodies against the SBP and GST tags. (**A**) LeishIF4E-5-GST interacts directly with LeishIF4G-1. The top panels represent the blot developed with anti-SBP antibodies while the bottom panels represent the blot developed with anti-GST antibodies. (**B**) LeishIF4E-5-GST interacts directly with LeishIF4G-2-SBP. The top panels represent the blot developed with anti-SBP antibodies while the bottom panels represent the blot developed with anti-GST antibodies.

We verified the direct interaction between LeishIF4E-5 and LeishIF4G-1 or LeishIF4G-2 using recombinant LeishIF4E-5 tagged with Glutatione sulfotranferase (GST) and expressed in bacteria, along with extracts of *Leishmania* cells that expressed either the SBP-tagged LeishIF4G-1 or LeishIF4G-2, and control cells that expressed the SBP-tagged luciferase (Figure 6). Extract of cells expressing the SBP-tagged LeishIF4G paralogs and the SBP-tagged luciferase were incubated with streptavidin-Sepharose beads and washed. The bound beads were then incubated with bacterial lysates containing LeishIF4E-5-GST (Figure S9), washed, and eluted with biotin. Samples from the supernatant, flow-through, wash and eluted fractions were subjected to Western analysis. The LeishIF4E-5 was monitored using antibodies against the GST tag, while LeishIF4G-1, LeishIF4G2 and luciferase were monitored using antibodies against the SBP tag. Figure 6A,B shows that LeishIF4E-5 coeluted with either LeishIF4G-1 or LeishIF4G-2 (left panels) but not with luciferase (right panels), in which LeishIF4E-5-GST was found only in the flow-through and washes, but not in the eluted fraction. In these pull-down assays, both LeishIF4G-1 and LeishIF4G-2 migrated faster than expected, possibly representing cleavage products. We also observed multiple bands of both proteins in the elution fractions, and these too were suspected to represent cleavage products of LeishIF4G-1 and LeishIF4G-2. To verify that these bands were derived from LeishIF4G-1 and LeishIF4G-2, we excised these bands from the gels and subjected them to mass spectrometry analysis. The results shown Figure S10A,B and Table S3 show that all the bands that were immunostained by the anti-SBP antibodies contained peptides derived from the LeishIF4G-1 and LeishIF4G-2, respectively, confirming that they represented fractions of LeishIF4G-1 and LeishIF4G-2. We mark here that other translation factors in *Leishmania* were reported to be highly susceptible to proteolytic cleavage, including Leish4E-IP1 and Leish4E-IP2 [28].

## 3. Discussion

*Leishmania* parasites are exposed to changing environmental conditions, requiring dedicated programs for gene expression that promote the adaptation of parasites to their altered environments. We also witness a multitude of cap-binding eIF4E isoforms that could assist the adaptation processes described, especially during stage transformation. Our group and others have characterized the putative functions and structure of eIF4E isoforms in *Leishmania* [29,30]. A less familiar eIF4E was identified in *Trypanosoma brucei*, TbIF4E-5 [21]. The knock-down of TbIF4E-5 by RNA interference (RNAi) resulted in impaired motility and a slight reduction in translation. In this study, we investigated the *Leishmania* (LeishIF4E-5) ortholog of TbIF4E-5 and examined its function during its life cycle.

Sequence analysis shows that LeishIF4E-5 is different from its mammalian counterpart and from other LeishIF4Es. It is also interesting to note that the three conserved aromatic residues in the LeishIF4E-5 cap-binding pocket and in its trypanosomatid orthologs contain two Trp residues and a conserved Tyr residue, unlike the canonical eIF4Es that all contain three Trp residues at positions that are parallel to the mouse 56, 102 and 166. The interacting partners of LeishIF4E-5 (LeishIF4G-1 and LeishIF4G-2) are also different from their mammalian counterparts and their corresponding LeishIF4G paralogs. These sequence variations highlight the evolutionary divergence of *Leishmania* and could indicate that the multiple paralogs observed for components of the cap-binding complex may have originated early during their evolution. It should be noted that the multiple paralogs of LeishIF4E and LeishIF4G are unique to these two components of the cap-binding complex, and are not typical of other translation factors, such as subunits of LeishIF3, orthologs of LeishIF2 subunits and other factors.

In an attempt to understand the potential role of LeishIF4E-5, it was overexpressed in transgenic parasites. The translation efficiency of cells that overexpressed LeishIF4E-5-SBP was lower than that observed for cells that overexpressed LeishIF4E-1-SBP, or the non-related CAT reporter gene (Figure 2). Although this assay does not directly monitor the translation potential of the investigated eIF4E but rather gives the general snapshot of the global translation at the given time, it does give an indication on the overall effect of overexpressing LeishIF4E-5, whether direct or indirect. A general decline in global translation which could be observed in control cell lines that express transgenic proteins as compared to wild type cells was well evident and has been previously reported for unrelated proteins too [14,17]. We hypothesize that this could be due to competition for the same pool of translation factors or chaperones, leading to a competitive block in translation. However, the reduction in the global translation of transgenic cells that over expressed LeishIF4E-5 was stronger and reached 67.6% as compared to the global translation in wild type cells. In line with the translation assay, we observed that LeishIF4E-5 overexpression had a profound effect on the cellular proliferation of parasite cells.

The cap-binding affinity of eIF4E is one of the factors that can influence translation efficiency. We previously showed that the different LeishIF4Es differ in their binding affinities with m^7^GTP and cap-4, with the highest in vivo affinity towards m^7^GTP exhibited by LeishIF4E-1 and LeishIF4E-4 [7], in accordance with their putative roles during translation in the cell. The cap-binding activity of eIF4E depends on the presence of conserved tryptophan or aromatic residues in their cap-binding pocket. LeishIF4E-1 exhibits the conservation of all the three homologous tryptophan residues at positions 56, 102 and 166 that represent positions in the mouse eIF4E. Exchanging one of these conserved residues can impair the cap-binding affinity, as observed for LeishIF4E-3, in which the Trp residue at position 170 is exchanged by Met [7]. Since the tryptophan at the position homologous to Trp102 in LeishIF4E-5 is substituted with Tyr, an aromatic residue, this substitution is expected to maintain the cap-binding activity. Indeed, LeishIF4E-5 retained its binding to m^7^GTP, but only in promastigotes. The binding was monitored by affinity purification assays over m^7^GTP-agarose and appeared to be lower than that observed for LeishIF4E-1 in promastigotes (Figure 4). For comparison, the cap-binding of TcIF4E-5 to the m^7^GTP and cap-4 analogs as determined by thermophoresis, indicated a low affinity of TcIF4E-5 to m^7^GTP and cap-4 in vitro (*K*_d_ = 21 ± 7 and 110 ± 33 μM, respectively) [22]. A relatively weak cap-binding activity was also observed for TbIF4E-5 [22], in accordance with our observations on LeishIF4E-5. In this study, we also show that the cap-binding activity of LeishIF4E-5 was restricted to the promastigote life form, through a mechanism yet unresolved. However, we hypothesize that this mechanism could be related to its putative post-translational modifications, which could be affected by the environmental conditions that lead to cell differentiation.

To further understand the function of LeishIF4E-5, we attempted to identify its interacting partners by pull-down assays and subsequent mass spectrometry analyses. The enriched proteins that co-precipitated with LeishIF4E-5 were subjected to manual classification and to the GO term enrichment analysis, which highlighted the association of LeishIF4E-5 with proteins classified as cytoskeletal, signaling, nuclear proteins, several ribosomal proteins, RNA helicases and RNA binding proteins. While most LeishIF4E paralogs copurify with a variety of RNA binding proteins, the finding of nuclear proteins is well coordinated with the observed LeishIF4E-5 foci that surround the nucleus. The enrichment of cytoskeletal and flagellar proteins that associate with LeishIF4E-5 suggests that the latter could have a role in the motility of the parasite, as also observed for the TbIF4E5 ortholog [21]. Further, the enrichment of cytosolic ribonucleoprotein components, including an exosome subunit (rrp6), suggests that LeishIF4E-5 could have a role in mRNA degradation, possibly in dedicated foci. The non-uniform distribution and its association with discrete foci was also supported by the confocal analysis. We searched for the *T. brucei* ortholog, TbIF4E-5, in the TrypTag database. TrypTag is a database that contains the localization of the majority of proteins encoded by the *T. brucei* genome [31]. In *T. brucei*, the analysis revealed that the majority of the proteins were homogenously localized in the cytoplasm, whereas a small portion concentrated in cytoplasmic granules. The association of ATP dependent RNA helicases and RNA binding proteins with LeishIF4E-5 suggests the possible role of these proteins in the remodeling of the ribonucleoprotein (RNP) particles. The enrichment of PUF1 previously has been reported in both *T. brucei* and *Leishmania* starvation-induced stress granules [15,32]. PUF1 usually functions as an important post-transcriptional regulator of RNA turnover [33]. A single subunit of exosome (rrp6) was shown to associate with LeishIF4E-5, and rrp6 is localized in the nucleus and involved in mRNA and rRNA quality control [34], suggesting that LeishIF4E-5 could interact with the RNA quality control machinery. Furthermore, the enrichment of the *Leishmania* ortholog of the *T. brucei* decapping enzyme TbALPH1 (LmjF.22.1600) with LeishIF4E-5 also points out that the LeishIF4E-5 could be associated with the RNA degradation machinery (Table S1). In *T. brucei,* TbALPH1 was identified in stress granules and was shown to carry out decapping activity of mRNAs [26]. In addition, the DEAD-box RNA helicase DHH1 (LmjF.35.0370) was also enriched with the LeishIF4E-5, possibly indicating the nature of the foci in which LeishIF4E-5 is found. The presence of numerous metabolic enzymes found enriched with LeishIF4E-5 is rather intriguing, but studies suggest that some metabolic enzymes could bind RNA even in the absence of canonical RNA binding motifs [25]. Lastly, the association of chaperones with LeishIF4E-5 is not surprising, as the expression of chaperones usually increases along with the overexpression of transgenic proteins.

Since LeishIF4E-5 could have a putative role in translation, it is essential to identify its LeishIF4G binding partner. eIF4G orthologs are part of a family of MIF4G domain proteins, some of which function as translation factors, and others as inhibitors of cap-dependent translation, such as DAP5 for example [35,36]. Therefore, the association with two different LeishIF4Gs could indicate that LeishIF4E-5 forms different complexes that are responsible for different functions. Our mass spectrometry data highlighted two LeishIF4G paralogs (LeishIF4G-1 and LeishIF4G-2) co-eluting with LeishIF4E-5. The direct interaction of LeishIF4E-5 with LeishIF4G-1 and LeishIF4G-2 was verified by recombinant interaction assays. This is in agreement with the results obtained in *T. brucei*, where both TbIF4G-1 and TbIF4G-2 interacted with the TbIF4E-5 [21]. In *T. brucei,* the presence of accessory proteins was found in the LeishIF4G-2 complex, emphasizing the functional difference between the two complexes [21]. The two LeishIF4Gs vary from each other, with respect to the conserved YXXXXLφ element, which is observed only in LeishIF4G-2 and not in LeishIF4G-1. LeishIF4G-1 varies from TbIF4G-1, as the latter does contain a putative conserved eIF4E-interacting domain, whereas LeishIF4G-1 lacks this element. With respect to LeishIF4G-2, the residues that occupy the 7th position of the suggested eIF4E-interacting element are maintained only at positions 1 and 6 (Tyr and Leu, respectively), while the residue at position 7 is not hydrophobic and not conserved. Such limited conservation was previously reported for the LeishIF4G-3 element, where a charged Asp residue occupies the 7th position, and residues found at positions 2–5 are significant for the interaction with LeishIF4E-4 [10]. We also observed that both the overexpressed LeishIF4G-1 and LeishIF4G-2 were subjected to degradation. This could imply that the expression of transgenic LeishIF4G-1 and LeishIF4G-2 is tightly regulated, and their overexpression disrupts this regulation. Thus, by degradation cells can regulate the levels of active LeishIF4G-1 and LeishIF4G-2. We therefore could not exclude the possibility that the degradation occurred due to the overexpression of both proteins.

Overall, the results indicate that LeishIF4E-5 could be part of cytoplasmic granules with distinct roles, as several granule components were observed in the mass spectrometry analysis. Further investigation is required to understand the mode of interaction between LeishIF4E-5 and either LeishIF4G-1 or LeishIF4G-2. In addition, although LeishIF4E-5 interacted moderately with the mRNA cap structure, m^7^GTP, the global translation of LeishIF4E-5 overexpressing cells remained low. Therefore, our data could indicate that LeishIF4E-5 may act as a translation repressor that moderately competes with other cap-binding factors on binding to the cap structure and reduces the accessibility of the mRNA cap to other translation factors in promastigotes. We also consider the possibility that post-translation modifications could alter its function under different environmental conditions.

## 4. Materials and Methods

### 4.1. Cell Culture

Promastigotes of *Leishmania (L.) amazonensis (L. amazonensis),* strain (MHOM/BR/LTB0016) promastigotes were grown at 25 °C in M199 medium (Biological Industries, Beit Haemek, Israel) supplemented with 10% heat-inactivated fetal calf serum (FCS, Biological Industries, Beit Haemek, Israel), HEPES pH 7.4 (Sigma, St. Louis, MO, USA), 10 mM adenine (Sigma, St. Louis, MO, USA), 4 mM L-glutamine (Biological Industries, Beit Haemek, Israel), 5 μg/mL hemin and 100 U/mL penicillin and streptomycin (Biological Industries, Beit Haemek, Israel). Experiments were performed with logarithmically growing parasites at a cell density of 4–7 × 10^6^ cells/mL.

### 4.2. Bioinformatics

The LeishIF4E-5 Open Reading Frame (ORF) from *L. amazonensis* (LAMA_000312000), *Trypanosoma brucei* (Tb927.10.5020), *Crithidia fasciculata* (CFAC1_250013200), *Leptomonas seymouri* (Lsey_0058_0010) and sequences of *L. amazonensis* eIF4Es, LeishIF4E-1 (LAMA_000544700), LeishIF4E-2 (LAMA_000303800), LeishIF4E-3 (LAMA_000580200), LeishIF4E-4 (LAMA_000590600), and LeishIF4E-6 (LAMA_000504800) were obtained from the TriTryp database [37]. In addition, the *L. amazonensis* and *T. brucei* sequences for eIF4Gs, LeishIF4G-1 (LAMA_000225900), LeishIF4G-2 (LAMA_000238000), LeishIF4G-3 (LAMA_000257600), LeishIF4G-4 (LAMA_000367400), LeishIF4G-5 (LAMA_000167100) and TbIF4G-1 (Tb927.5.1490), TbIF4G-2 (Tb927.9.5460), TbIF4G-3 (Tb927.8.4820) were also obtained from the TriTryp database. The protein sequences of the canonical eIF4E from *Homo sapiens* (P06730), *Mus musculus* (P63073), *Saccharomyces cerevisiae* (P07260), *Plasmodium falciparum* (O97266) and eIF4G-1 (Q6NZJ6) were obtained from the NCBI Protein database [38]. The canonical eIF4E of *Caenorhabditis elegans* (IFE-3, CE17331) was obtained from the WormBase database. Multiple sequence alignment was performed using Clustal Omega and color alignment conservation was created using Jalview for multiple sequence alignment editing, visualization and analysis [39]. The eIF4E sequences were multiple-aligned using ClustalW of MEGA X software (Molecular Evolutionary Genetic Analysis version 10.2.4) with its default options [40,41]. The phylogenetic tree was constructed using Neighbor Joining (NJ) and BioNJ with the Jones–Taylor–Thornton model distance matrix. The phylogenetic tree is drawn to scale, with branch lengths measured in the number of substitutions per site without bootstrapping.

### 4.3. Plasmid Construction

The streptavidin binding protein (SBP, 6 kDa) was used to tag the C-terminus of LeishIF4E-5 (645bp), LeishIF4G-1 (3048bp) or LeishIF4G-2 (4362bp). The LeishIF4E-5, LeishIF4G-1 and LeishIF4G-2 ORFs were PCR amplified from genomic DNA using the primers derived from the relevant genes. LeishIF4E-5 derived primers were forward 5′-gctggatccATGTCGGCCACACACGC-3′ and reverse 5′-gctctagaACAATCCTTCACCATG-3′. LeishIF4G-1 derived primers were forward 5′-gctggatccATGCTGATGGAAACACAG-3′ and reverse primer 5′-gctctagaCGATAAGTATGTGAGG-3′. LeishIF4G-2 derived primers were forward 5′-gctggatccATGGAGCACTCCTGTCGTG-3′ and reverse 5′-gctctagaACGGGCAGACTTTGCAC-3′. The LeishIF4E-5, LeishIF4G-1 and LeishIF4G-2 ORFs were cloned into the BamHI and XbaI restriction sites of the pX-H-SBP-H plasmid cassette, between two intergenic regions (H) derived from the Hsp83 genomic locus [11] that provide the signals for mRNA processing of the transgene. The resulting plasmid was denoted pX-H-4E5-SBP-H, pX-H-4G1-SBP-H or pX-H-4G2-SBP-H. *L. amazonensis* cells were transfected with the plasmid as described previously [42]. Stably transfected parasites were selected by their resistance to G418 (200 μg/mL).

### 4.4. Promastigotes Growth Analysis

Growth was monitored for promastigotes of *L. amazonensis* WT cells, transgenic parasites expressing the SBP-tagged LeishIF4E-5 and the SBP-tagged LeishIF4E-1, along with control cells expressing the chloramphenicol acetyltransferase (CAT) reporter gene. Cells were seeded at a density of 1 × 10^6^ cells/mL and grown for 5 days in complete medium. Cells were fixed and counted daily in triplicates.

### 4.5. Differentiation of Promastigotes to Axenic Amastigotes

Host free differentiation of transgenic *L. amazonensis* promastigotes expressing SBP-tagged LeishIF4E-5 to amastigote-like cells was performed in M199 media supplemented as described above, with a pH that was adjusted to 5.5 with sodium succinate. Log-phase promastigotes were transferred to acidic M199 and allowed to grow at 33 °C while gently shaking. Differentiated cells were tested 4 days following transfer to the acidic medium and increased temperature.

### 4.6. Confocal Microscopy

Transgenic *L. amazonensis* promastigotes expressing the SBP-tagged LeishIF4E-5 were grown in an 8 well μ-Slide (Ibidi GmbH, Gräfelfing, Germany) until log phase. Cells were washed once in serum-free M199 and fixed in 2% paraformaldehyde for 30 min at room temperature followed by a wash with 1× phosphate buffered saline (PBS). Fixed cells were stained using an antibody against the SBP tag (Milipore, 1:100) and a secondary anti-mouse IgG DyLight 488 (3:500) (KPL, Milford, CT, USA). DNA was counterstained using 4’, 6-diamidino-2-phenylindole (DAPI). Images were recorded using an inverted Zeiss LSM 880 Axio-observer Z1 confocal laser scanning microscope with Airyscan detector. The cells were observed under Plan-Apochromat 63×/1.4 oil, 1.4 NA, DIC objective. Images were acquired in 512 × 512 pixels format with 8× digital zoom (1.8× for broad field) using Zen lite software (Carl Zeiss AG, Oberkochen, Germany). Images were processed using the Image J software package [43] and a representative image is presented.

### 4.7. Translation Assay

Global translation was measured using the SUrface SEnsing of Translation (SUnSET) method [44,45]. Puromycin is a structural analog of tRNA that enters the A site where it transfers to the nascent polypeptide chain causing a premature chain release [46,47]. This method monitors de novo protein synthesis at the time of puromycin incorporation. Transgenic parasites expressing the SBP-tagged LeishIF4E-5, LeishIF4E-1 or control cells expressing the CAT reporter gene along with WT cells were incubated with 1 μg/mL puromycin for 20 min. Cells were then washed with PBS and with post-ribosomal sup with supplements (PRS^+^, 35 mM HEPES, 100 mM KCl, 10 mM MgCl_2_, 20 mM NaF, 50 mM β-glycerophosphatase, 2× protease inhibitors, 2 mM iodoacetamide and 1 mM dithiothreitol, DTT) buffer before lysis in Laemmlli’s buffer. Cell lysates (equal protein loads) were resolved over 12% SDS-PAGE and subjected to Western analysis using an antibody against puromycin (DSHB, University of Iowa, 1:1000). Parasites were also exposed to cycloheximide (100 μg/mL) (Sigma, MO, USA) before incubation with puromycin as a negative control for the assay.

### 4.8. In Vivo Pull-Down of Tagged LeishIF4E-5

Transgenic parasites expressing the SBP-tagged LeishIF4E-5 (200 mL) were grown until their mid log phase, washed twice with ice-cold PBS, once with PRS^+^ buffer and lysed in PRS^+^ containing 1% Triton X-100. Lysed cells were clarified by centrifugation at 20,000 *g* for 20 min in 4 °C. A supernatant aliquot (S) was saved as an input for the pull-down assay. Cell extracts were incubated with streptavidin-Sepharose beads (GE Healthcare, Buckinghamshire, UK) for two hours. Following binding, the flow-through fraction (FT) was collected, and the beads were washed twice with 0.1% NP-40 containing PRS^+^ (W). Bound LeishIF4E-5 complexes were eluted from the beads by incubation with PRS^+^ supplemented with 0.1% NP-40 and 5 mM biotin (E). The eluates were precipitated with 10% trichloro-acetic acid (TCA), washed once with acetone and dissolved in 5× Laemmli’s buffer. An aliquot of the eluted protein sample was resolved over 12% SDS-PAGE and subjected to Western analysis using antibodies against proteins of interest: the SBP tag, LeishIF4E-1 or LeishIF4E-4. The remaining fraction of the eluted proteins was subjected to mass spectrometry analysis.

### 4.9. In Vitro Cap-Binding Assay

Transgenic *L. amazonensis* promastigotes expressing the SBP-tagged LeishIF4E-5 were harvested, washed twice in PBS and once with column binding buffer (CBB: 20 mM HEPES pH 7.6, 2 mM EDTA, 50 mM NaCl and 1 mM DTT). Cells were lysed in CB^+^ (CB supplemented with phosphatase inhibitors: 20 mM sodium fluoride, 50 mM β-glycerophosphatase, 2 mM iodoacetamide and 2× protease inhibitor) and 1% Triton X-100. Extracts were clarified by centrifugation at 20,000 *g* for 20 min in 4 °C. A fraction from the supernatant was saved as input (S). The clarified cell extract was incubated with m^7^GTP-agarose beads (Jena Biosciences, Jena, Germany), pre-calibrated with CBB^+^. Following binding, the flow-through (FT) fraction was collected. Beads were then washed twice with CBB^+^ and once with CB^+^ supplemented with 100 μM GTP (W). Bound proteins were eluted (E) using CB^+^ supplemented with 200 μM m^7^GTP followed by 10% TCA precipitation followed by an acetone wash, resuspended in Laemmli’s sample buffer and resolved over 12% SDS-PAGE. The protein complexes were subjected to Western analysis using monoclonal antibodies against the SBP tag or LeishIF4E-1 as a positive control. The assay was repeated with *L. amazonensis* axenic amastigotes expressing the SBP-tagged LeishIF4E-5. The bands observed in the Western blots were quantified using Multi Gauge version 2.0.

### 4.10. Mass Spectrometry Analysis

#### 4.10.1. Sample Preparation

The eluted protein complexes were resolved on 12% SDS-PAGE. Gels were stained with Coomassie blue and mass spectrometric analysis was performed on triplicate samples in the Smoler Proteomics Center, Technion, Israel. 

#### 4.10.2. Mass Spectrometry

Proteins were separated on SDS-PAGE and were further reduced using 3 mM DTT (60 °C for 30 min) followed by modification with 10 mM iodoacetamide in 100 mM ammonium bicarbonate for 30 min, at room temperature. The proteins were then subjected to over-night digestion in 10 mM ammonium bicarbonate in trypsin (Promega, Madison, WI, USA) at 37 °C. Trypsin digested peptides were desalted, dried and re-suspended in 0.1% formic acid. Trypsinated peptides were resolved by reverse phase chromatography over 30 min linear gradient with 5% to 35% acetonitrile and 0.1% formic acid in the water, 15 min gradient with 35% to 95% acetonitrile and 0.1% formic acid in water and 15 min gradient at 95% acetonitrile and 0.1% formic acid in water at 0.15 µL/min flow rate. The mass spectrometric analysis was performed using a Q-Exactive Plus mass spectrometer (Thermo Fischer Scientific, Waltham, MA, USA) in positive mode repetitively full MS scan followed by High energy Collision Dissociation (HCD) of the 10 most dominant ions selected from the first MS scan. A mass tolerance of 10 ppm for precursor masses and 20 ppm for the fragment ions was set. 

#### 4.10.3. Statistical Analysis for Enriched Proteins

Raw mass spectrometric data were analyzed using the MaxQuant software, version 1.5.2.8 [48]. The data were searched against annotated L. major Friedlin proteins listed in the TriTryp database [37]. Protein identification was set at less than a 1% false discovery rate. The MaxQuant settings selected were a minimum of 1 razor/unique peptide for identification, a minimum peptide length of six amino acids and a maximum of two mis-cleavages. For protein quantification, summed peptide intensities were used. Missing intensities from the analyses were substituted with values close to baseline only if the values were present in the corresponding analyzed sample. The log_2_ of iBAQ intensities [49] were compared between the three SBP-LeishIF4E-5 biological repeats and the three SBP-luciferase repeats on the Perseus software platform [50], using a *t*-test. Adjusted P (Padj) values were corrected using permutation-based false discovery rate (FDR) = 0.05 and the number of randomizations = 250. These are marked as Padj [50]. The enrichment threshold was set to a log_2_-fold change ≥ 1.6 and *p* value ≤ 0.05. 

#### 4.10.4. Categorization of Enriched Proteins

The annotated proteins were first categorized manually, and the summed relative intensities for each category were shown. The annotated proteins were then categorized by the Gene Ontology (GO) annotation via TriTrypDB, based on cellular components. The threshold for the calculated enrichment of proteins based on their GO terms was set for three-fold, with a *p* value ≤ 0.01. This threshold eliminated most of the general groups that represented parental GO terms. GO terms for which only a single protein was annotated were filtered out as well.

### 4.11. Interaction between Recombinant LeishIF4E-5-GST and LeishIF4G-1-SBP or between LeishIF4E-5-GST and LeishIF4G-2-SBP

#### 4.11.1. Cloning

LeishIF4E-5 (645bp) was amplified by PCR from the *L. amazonensis* genomic DNA using the forward primer 5′-gctgaattcATGTCGGCCACACACGCAC-3′ and reverse primer 5′-gctctagaCTAATCCTTCACCATGGGCG-3′, where upper case letters represent the LeishIF4E-5 sequences and lower case letters represent restriction sites. Restriction sites for EcoRI and XbaI were introduced into LeishIF4E-5 amplicon to allow fusion with the glutathione sulfotransferase (GST) gene in the pGST-parallel expression plasmid [51]. The GST-tag was fused at the N-terminus of LeishIF4E-5 and expressed in *E. coli* BL21 cells.

#### 4.11.2. Expression of LeishIF4E-5-GST

The transformed BL21 *E. coli* cells expressing the GST tagged LeishIF4E-5 were grown at 37 °C until OD_600_ = 0.4. Expression was then induced by the addition of 0.5 mM Isopropyl-β-d-1-thiogalactopyranoside (IPTG) followed by incubation at 18 °C for 14–16 h.

#### 4.11.3. Interaction Assay

*L. amazonensis* cells (100 mL) expressing SBP-tagged LeishIF4G-1 or LeishIF4G-2 were seeded at a density of 4–6 × 10^6^ cells/mL, washed twice with PBS and once with disruption buffer (DB, 20 mM Tris-HCl pH 8.0, 200 mM NaCl, 1 mM EDTA, 5 mM MgCl_2_ and 5% glycerol) and resuspended in the same buffer supplemented with a cocktail of protease inhibitors (Sigma) and 2 mM iodoacetamide, along with phosphatase inhibitors: 20 mM sodium fluoride, 50 mM β-glycerophosphatase, and 0.1% NP-40 (DB+). The cells were then lysed by the addition of 1% Triton X-100 for 10 min on ice and further centrifuged at 20,000 *g* for 20 min at 4 °C. A lysate sample was taken as supernatant (S). The clarified cell lysate was incubated with streptavidin-Sepharose beads (75 μL, GE Healthcare) that were pre-equilibrated by three washes of DB+ buffer for 2 h at 4 °C with constant shaking. The beads were then washed three times with DB+. In parallel, 200 mL of BL21 *E. coli* cells expressing the GST-tagged LeishIF4E-5 was washed twice with PBS, once with DB and resuspended in DB+. The bacterial cells were then disrupted twice in a French Press apparatus at 1500 psi. The lysed cells were centrifuged at 45,000 rpm in a Ti70 rotor (Beckman Coulter) for 45 min. The bacterial lysate (10 mL) was then incubated with the streptavidin-Sepharose beads on which LeishIF4G-1 or LeishIF4G-2 was pre-immobilized, for 2 h at 4 °C with constant shaking. A flow-through fraction was collected post binding. The beads were then washed five times with 1ml of DB+ and eluted with 5 mM of biotin in 1 mL DB+. Aliquots derived from the supernatant (S, 2%), flow-through (FT, 2%), wash (W, 25%) and eluted fractions (E, 25%) were resolved over 12% SDS-PAGE and further subjected to Western analysis using antibodies against the SBP tag (Millipore, Ternecula, CA, USA) to identify LeishIF4G-1 or LeishIF4G-2 and against the GST tag to identify LeishIF4E-5.

## Data Availability

All data sets are provided in the manuscript.

## Supplementary Materials

The following are available online at https://www.mdpi.com/article/10.3390/ijms22083979/s1, Figure S1: Sequence similarity of *L. amazonensis* LeishIF4E-5 with other paralogs. Figure S2: A phylogenetic tree that includes LeishIF4E-5 and its trypanosomatid orthologs, with different eIF4E-1 orthologs. Figure S3: Densitometry analysis of global translation in transgenic cell lines. Figure S4: A field view showing localization of SBP-tagged LeishIF4E-5. Figure S5: m^7^GTP binding of transgenic SBP-LeishIF4E-5 as compared to SBP-LeishIF4E-1. The cap-binding activity was evaluated by the Elution/Supernatant ratios. Figure S6: Sequence alignment of the putative LeishApaH-like phosphatase with TbALPH1 and the bacterial ApaH. Figure S7A: Various *Leishmania amazonensis* eIF4G sequences showing the presence or absence of the Y_XXXX_Lφ motif. Figure S7B: *T. brucei* eIF4G sequences showing the presence of the Y_XXXX_L motif. Figure S8: Sequence similarity of *L. amazonensis* LeishIF4G-1 and LeishIF4G-2 with different eIF4G candidates (4Gs). Figure S9: Expression of GST-tagged LeishIF4E-5 in bacteria. Figure S10. LeishIF4G-1 and LeishIF4G-2 are susceptible to proteolytic cleavage. Table S1: Manual categorization of proteins enriched in the LeishIF4E-5 interactome. Table S2: Identified proteome found enriched with LeishIF4E-5—classification by GO term enrichment. Table S3: Mass spectrometry analysis of peptides found in the different bands obtained from eluted LeishIF4G-1 and LeishIF4G-2 fractions.
