# Supplementary material for: LeishIF4E-5 Is a Promastigote-Specific Cap-Binding Protein in Leishmania
Source: Int J Mol Sci. 2021 Apr 12;22(8):3979. doi: 10.3390/ijms22083979 (PMC8069130; doi:10.3390/ijms22083979)
Supplement: Supplementary file 1 [file ijms-22-03979-s001.zip › CaptionsForSupFigs.docx]

**Supplementary Materials**


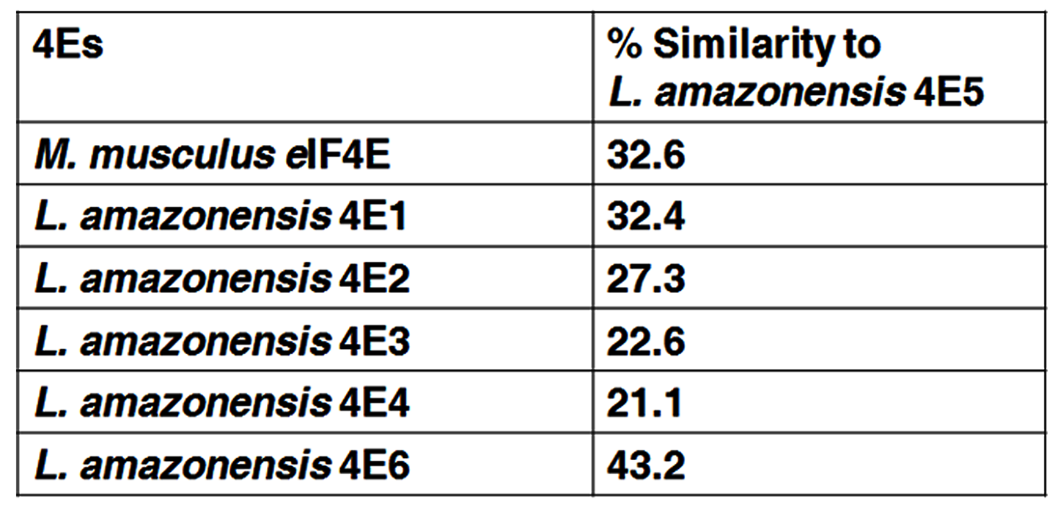


**Figure S1: Sequence similarity of *L. amazonensis* LeishIF4E-5 with other paralogs**- The table shows percent similarities between the LeishIF4E-5 and different *Leishmania* LeishIF4Es, and the *Mus Musculus* eIF4E.


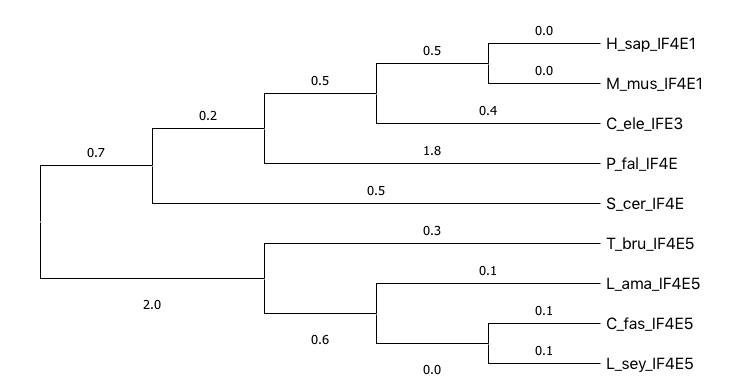


**Figure S2: A phylogenetic tree that includes LeishIF4E-5 and its trypanosomatid orthologs, with different eIF4E-1 orthologs.** Phylogenetic analysis of eIF4E from *H. sapiens, M. musculus, S. cerevisiae, C. elegans*, *L. amazonensis, T. brucei, C. fasciculata, L. seymouri* and *P. falciparum* by the Maximum Likelihood approach. Initial tree for the heuristic search was obtained by applying the Neighbor-join and BioNJ algorithm to a matrix of pairwise distances estimated using Jones-Taylor-Thorton model. The tree is drawn to scale, with branch lengths measured in the number of substitutions per site (the number of changes or ‘substitutions’ divided by the length of the sequence).


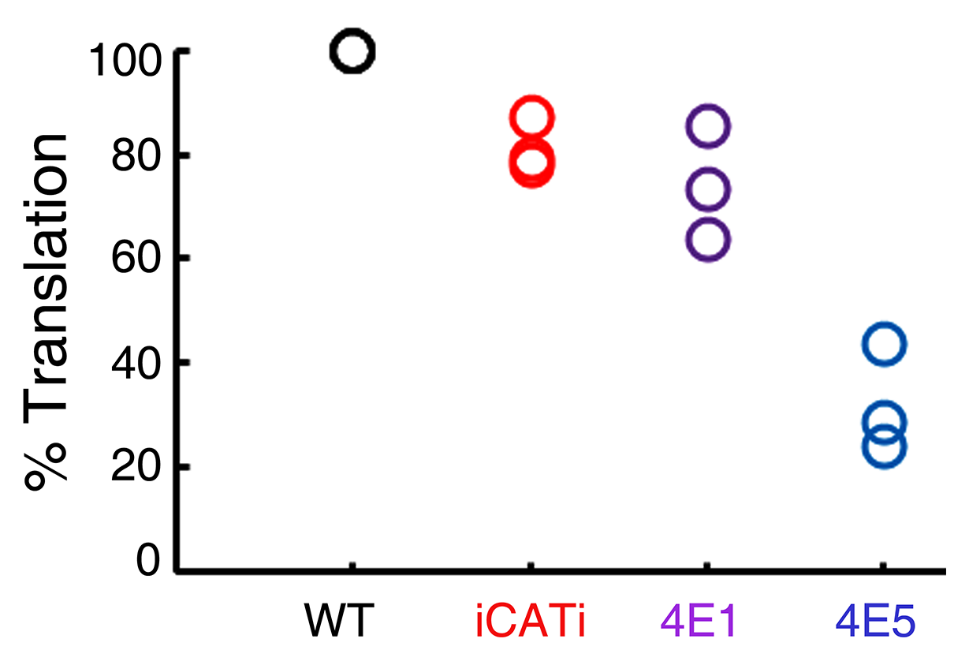


**Figure S3. Densitometry analysis of global translation in transgenic cell lines.**  Global translation was monitored under normal conditions, and the analysis was performed on each lane of the western blot shown in **Figure 2B.** Each lane was fully quantified using the Multi-Gauge software version 2.0. The values were normalized to the protein loads and presented as dot plots.


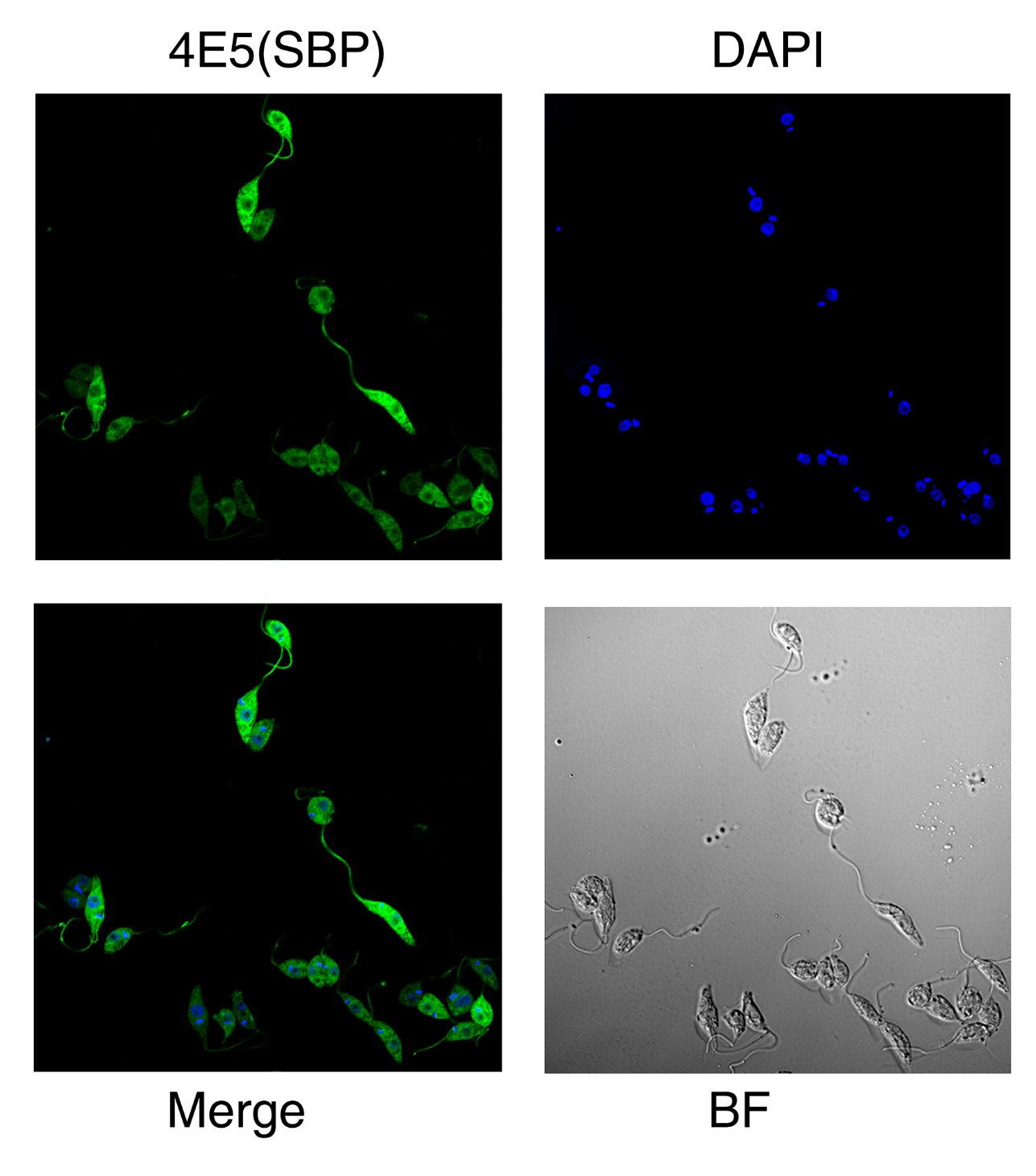
**Figure S4. A field view showing localization of SBP-tagged LeishIF4E-5.** Transgenic *L. amazonensis* promastigotes expressing SBP-LeishIF4E-5 were fixed, permeabilized and processed for confocal microscopy. SBP-LeishIF4E-5 was detected using a specific antibody against the SBP tag and a secondary DyLight-labeled antibody (488 nm; green). Nuclear and kinetoplast DNA were stained using DAPI (blue) and a bright field (BF) picture of the cells is also shown. The confocal analysis was repeated three times.


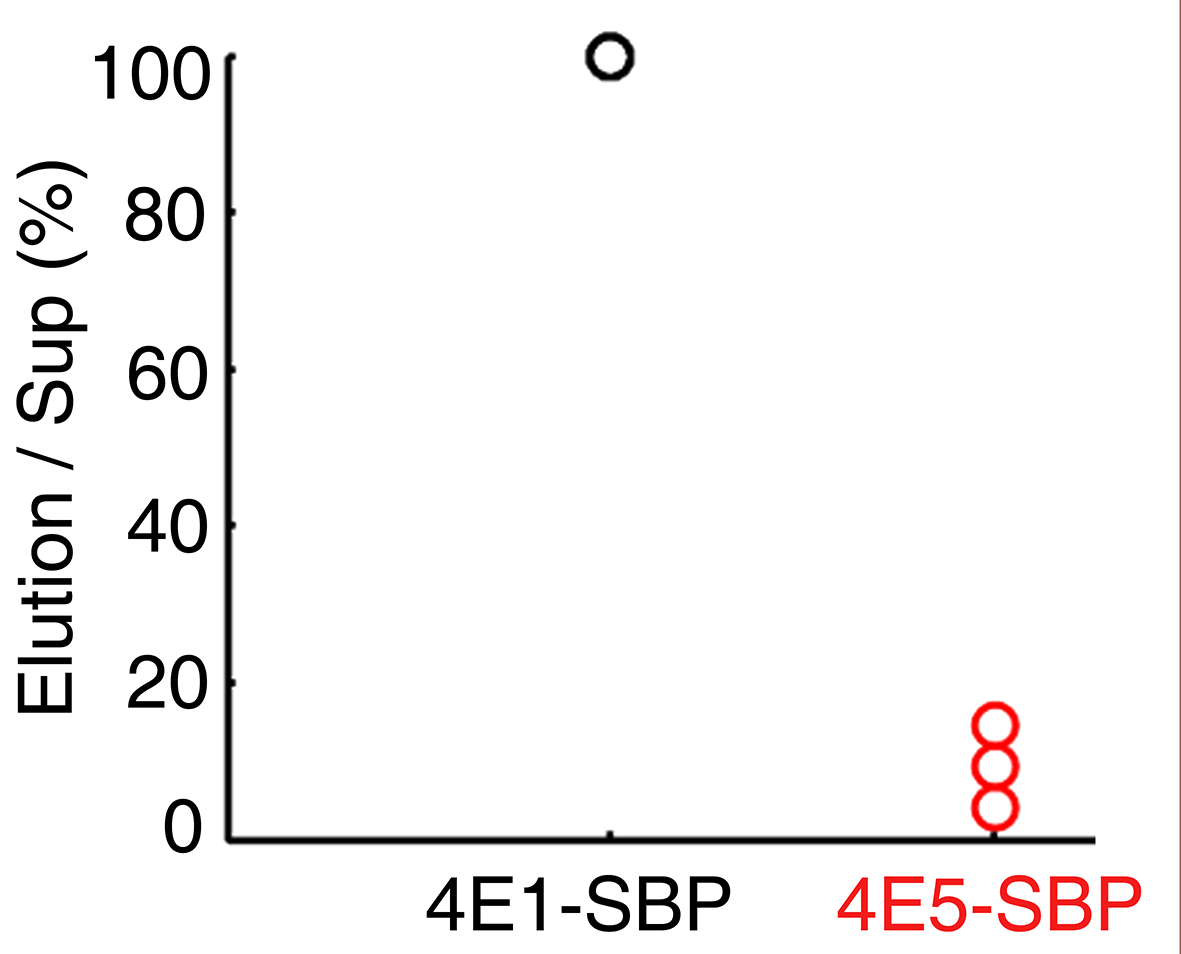


**Figure S5. m^7^GTP binding of transgenic SBP-LeishIF4E-5 as compared to SBP-LeishIF4E-1. The cap-binding activity was evaluated by the Elution/Supernatant ratios.** The dot plots represent the ratio of eluted proteins to their corresponding total loads. The ratio of eluted proteins in SBP-LeishIF4E-1 (black) and SBP-LeishIF4E-5 (red) were quantified using the Multi Gauge software version 2.0. Densitometry analysis was performed on three independent experiments.


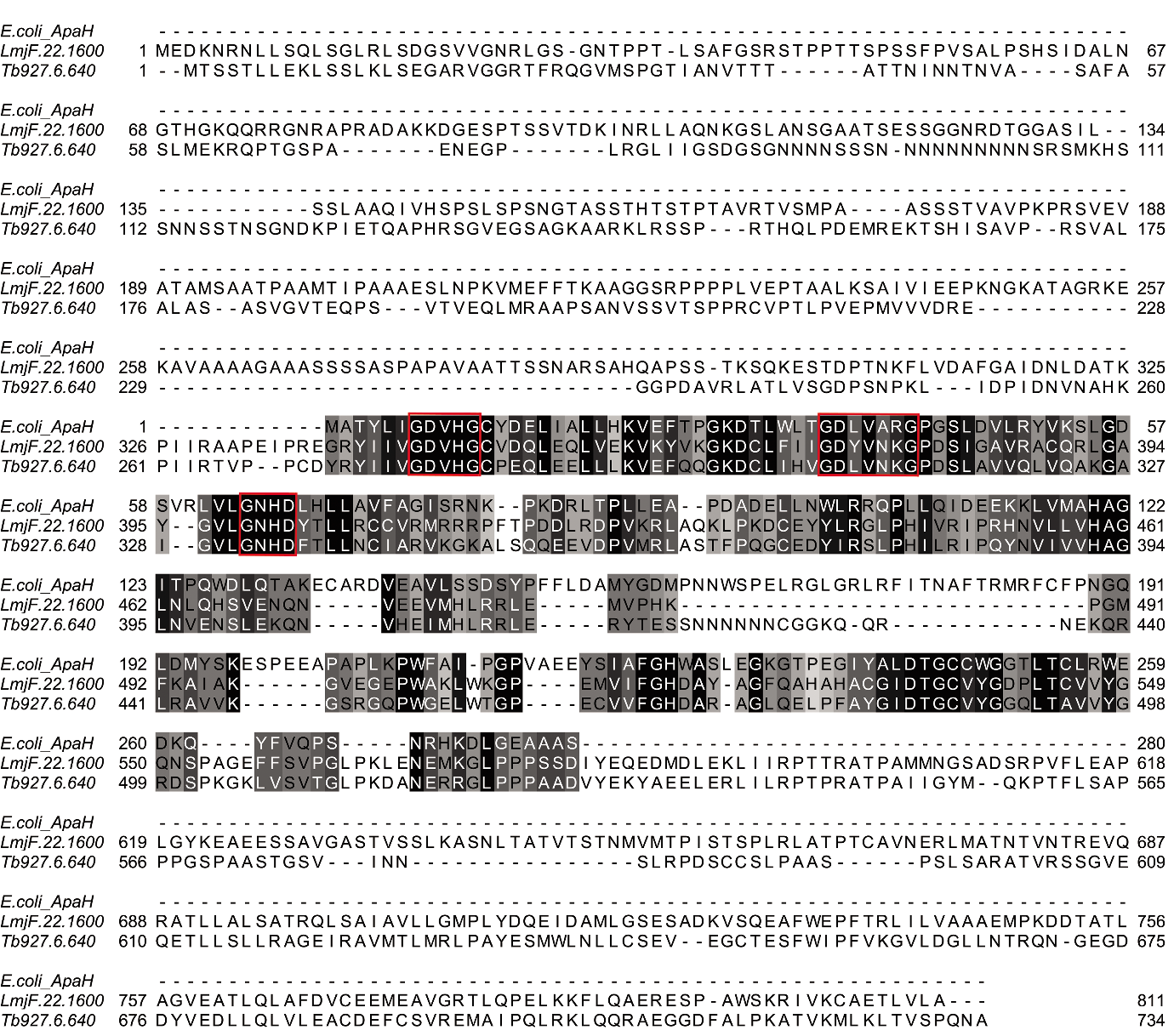


**Figure S6. Sequence alignment of the putative LeishApaH-like phosphatase with TbALPH1 and the bacterial ApaH.** Alignment of the signature motifs of TbALPH1 (Tb927.6.640, an ApaH-like phosphatase), with the ApaH of *E. coli* and the putative *L. major* ortholog LmjF.22.1600. The three catalytic signature motifs of the protein phosphatases (GDXHG, GDXXDRG and GNHE/D) are highlighted in red.


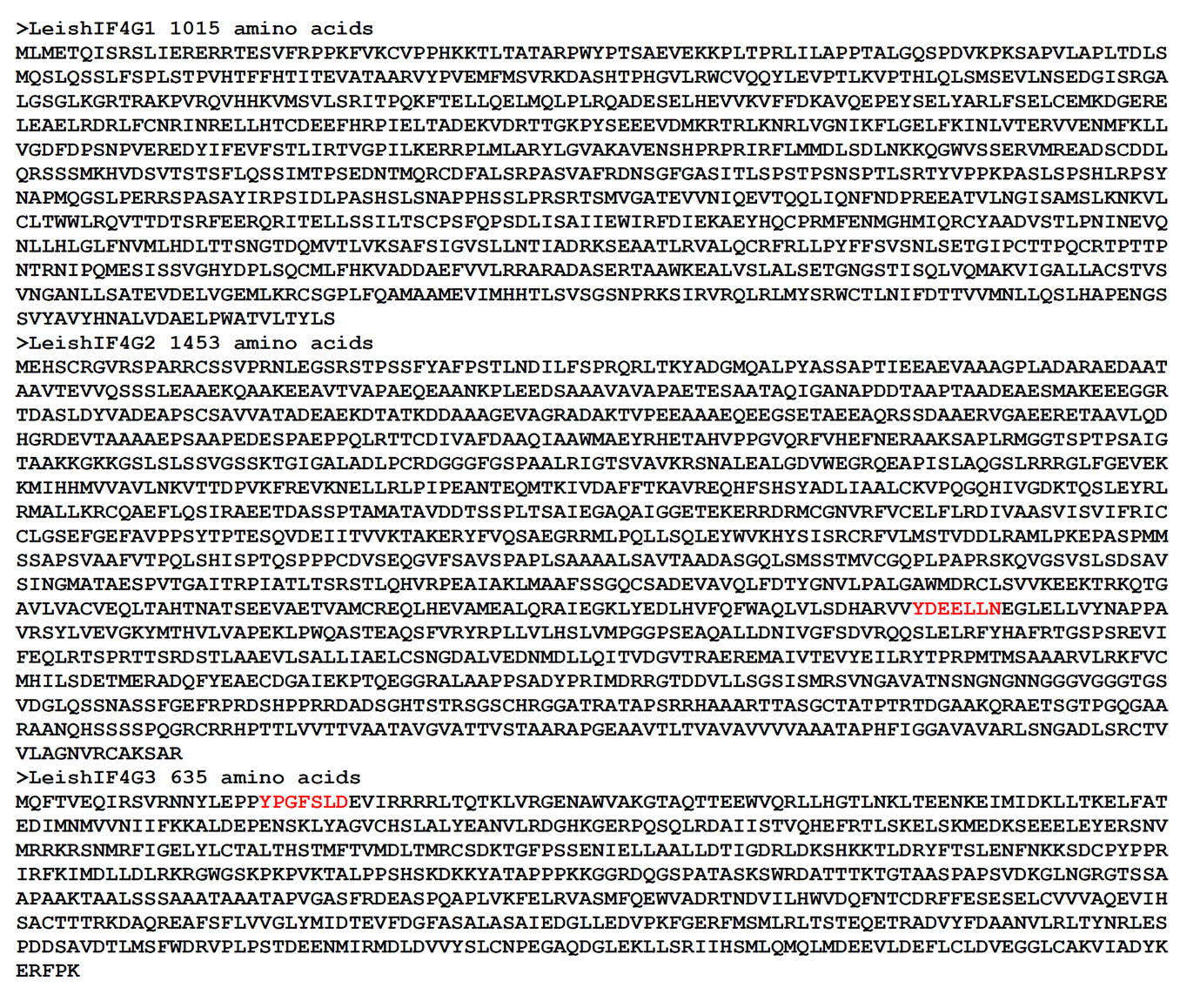
**Figure S7A.** **Various *Leishmania amazonensis* eIF4G Sequences showing the presence or absence of the Y_XXXX_Lφ motif**. The Y_XXXX_Lφ motif is required for binding to theLeishIF4E protein. Y refers to Tyrosine, X refers to any amino acid and L refers to Leucine. The φ residue represents a hydrophobic residue, but it is not conserved in *Leishmania.* The Y_XXXX_L is present in LeishIF4G-2 and LeishIF4G-3 while it is absent in LeishIF4G-1.


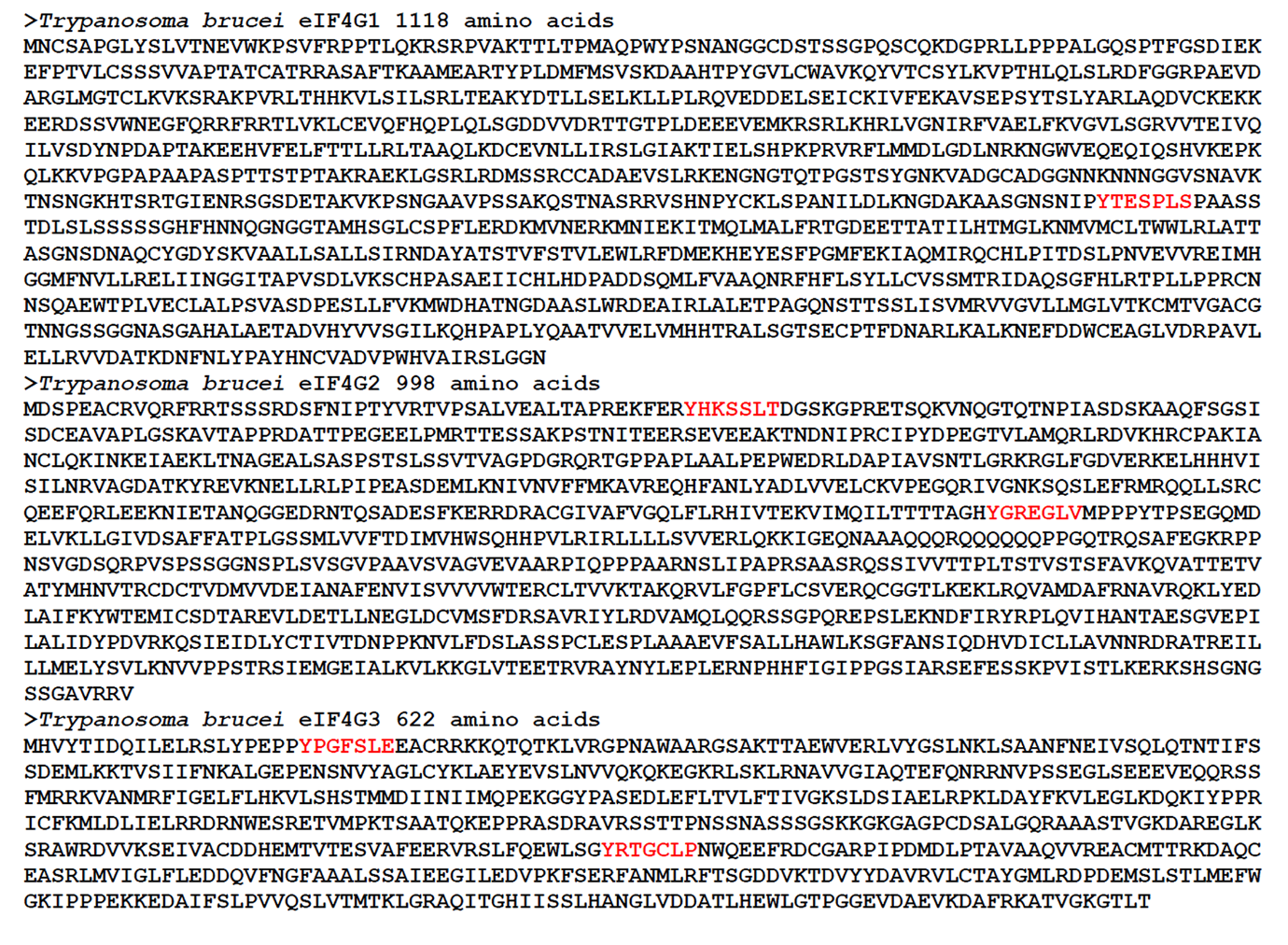
**Figure S7B.** ***T. brucei* eIF4G sequences showing the presence of the Y_XXXX_L motif**. The Y_XXXX_Lφ motif is required for binding to eIF4E proteins. Y refers to Tyrosine, X refers to any amino acid and L refers to Leucine. The φ residue represents a hydrophobic residue, but it is not conserved in trypanosomatids.


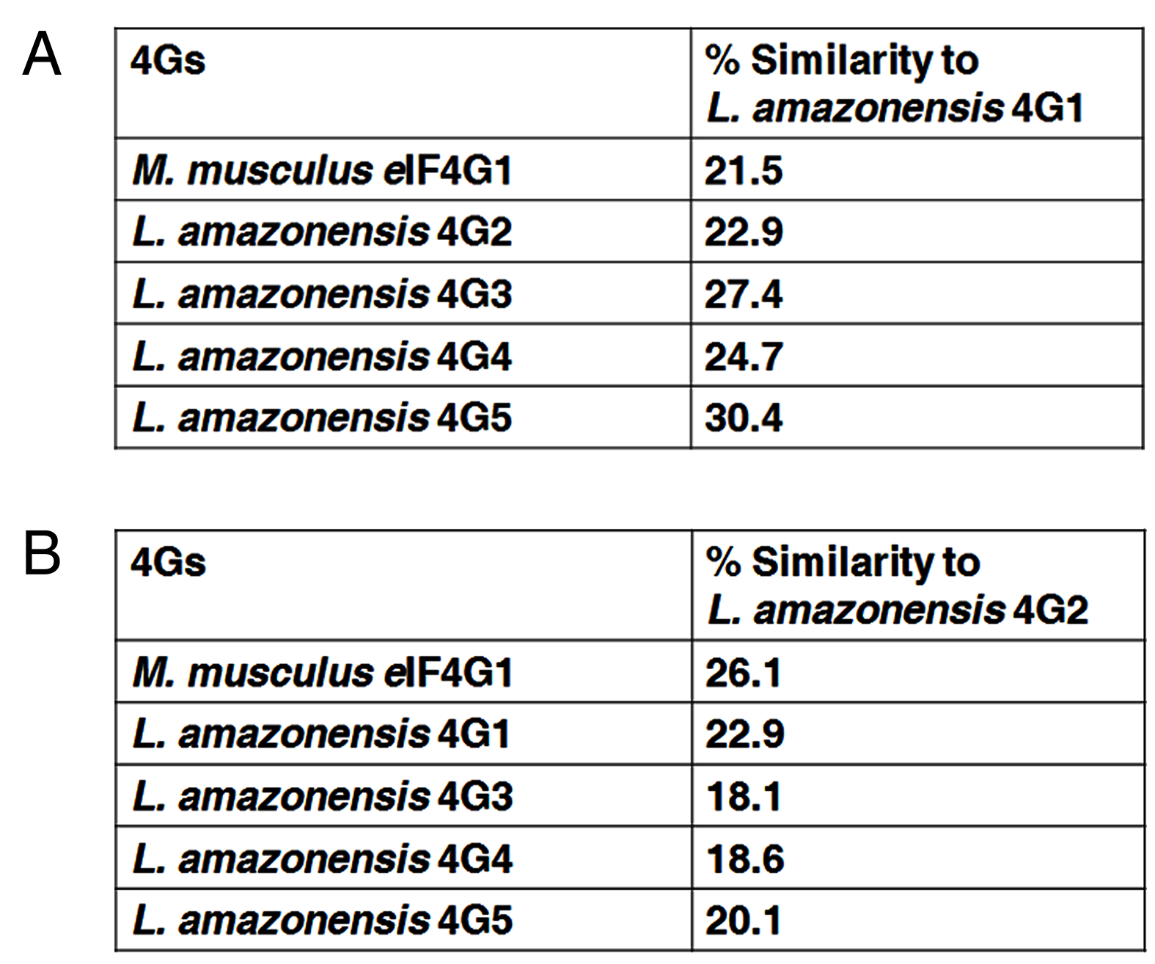


**Figure S8.** **Sequence similarity of *L. amazonensis* LeishIF4G-1 and LeishIF4G-2 with different eIF4G candidates (4Gs). (A)** The table shows the percent similarities between LeishIF4G-1 and different *Leishmania* LeishIF4Gs, as well as with the *Mus Musculus* eIF4G-1 **(B)** The table shows percent similarities between LeishIF4G2 and different *Leishmania* LeishIF4Gs, as well as with the *Mus Musculus* eIF4G-1. Percent similarities were generated by EMBOSS needle (<https://www.ebi.ac.uk/Tools/psa/emboss_needle/>).


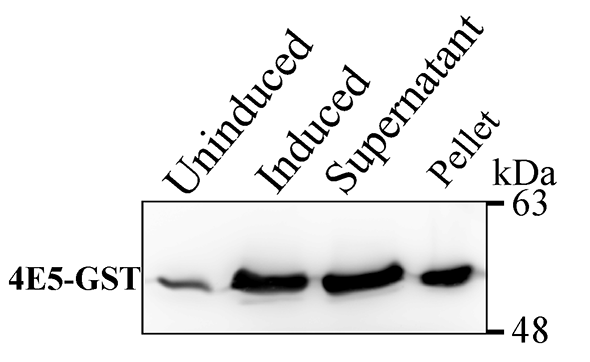


**Figure S9. Expression of GST-tagged LeishIF4E-5 in bacteria.** Full-length GST-tagged LeishIF4E-5 was expressed in BL-21 cells. Expression was induced by incubation with 0.5 mM IPTG. Cells were disrupted in a French Press, and the supernatant and pellet fractions were separated by centrifugation, to evaluate the solubility of the recombinant protein. Aliquots from the total extracts of uninduced and induced cells along with samples from the supernatant (Supernatant) and pellet fractions were resolved over SDS-PAGE and further subjected to western analysis using anti-GST antibodies.


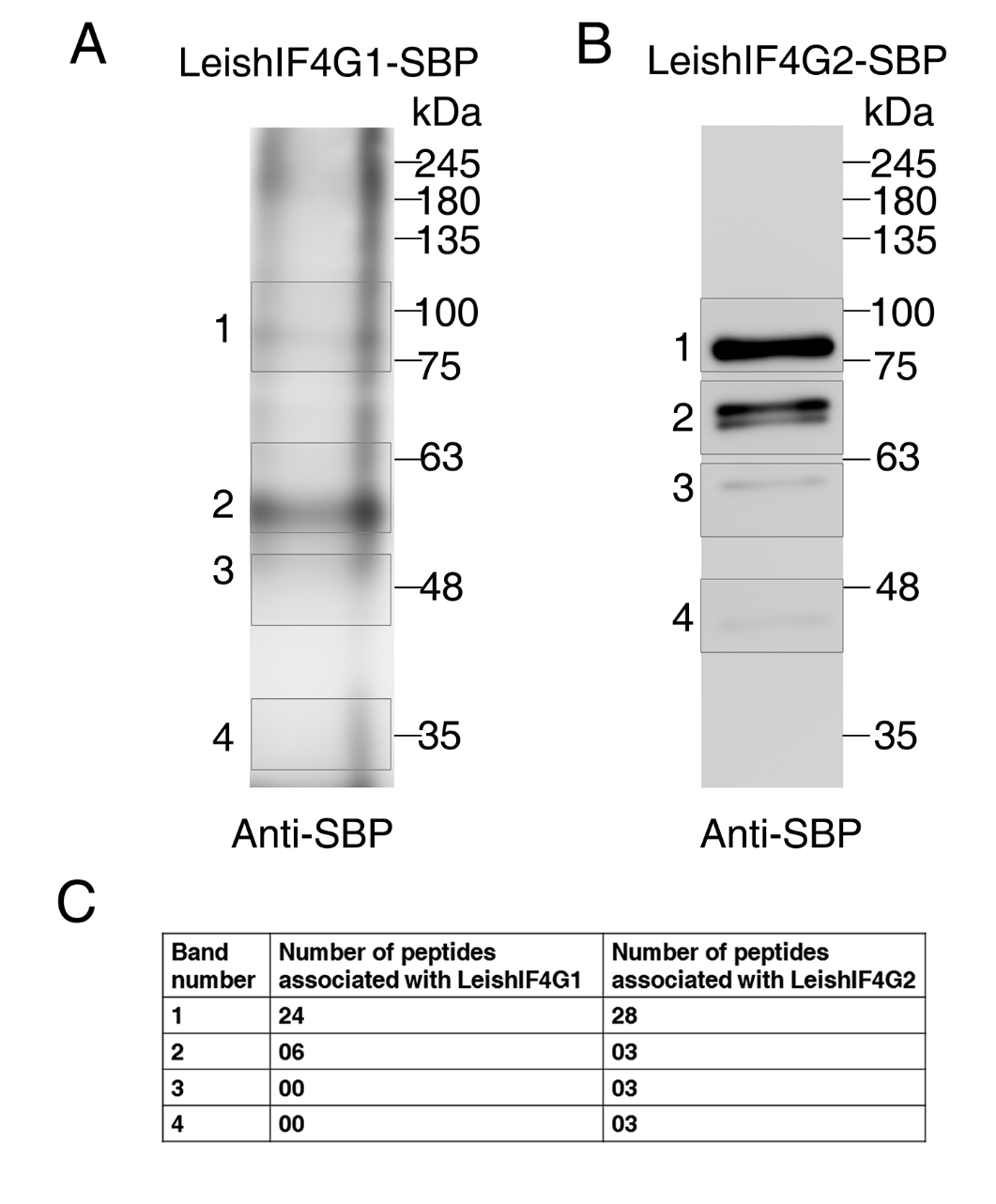
**Figure S10. LeishIF4G-1 and LeishIF4G-2 are susceptible to proteolytic cleavage**. Lysates of *L. amazonensis* expressing SBP-tagged LeishIF4G-1 **(A)** and LeishIF4G-2 **(B)** were purified over Streptavidin columns. The eluted fractions were separated over 10% SDS-PAGE gel and subjected to western analysis using monoclonal antibodies raised against the SBP tag. The bands that were highlighted by anti-SBP antibodies were excised from a parallel gel and subjected to Mass Spectrometry (MS) analysis, in search of peptides derived from the corresponding LeishIF4G. (**C)** The table shows the number of peptides obtained in the MS analysis of each band. The original MS data are given in Table S3.

**Table S1. Manual categorization of proteins enriched in the LeishIF4E-5 interactome.** The LeishIF4E-5 associated proteins were enriched by pull-down analysis of transgenic cells exptracts expressing SBP-tagged LeishIF4E-5 over streptavidin-Sepharose beads. The proteomic content was assessed by LC-MS/MS. Proteins were identified using MaxQuant software. Their enrichment as compared to the luciferase control, was determined using the Perseus statistical tool, setting the log_2_ fold change ≥1.6, with an adjusted P value (Padj)≤ 0.05 as the threshold (sheet 1). Identified proteins that were enriched with LeishIF4E-5 were categorized into groups according to their function. Raw data are shown in Sheet one, the filtered proteins above the threshold are shown in sheet 2 and their categorzation is given in sheet 3.

**Table S2.** **Identified proteome found enriched with LeishIF4E-5 – classification by GO term enrichment.** The LeishIF4E-5 associated proteins were identified as described in the legend of Table 1. The proteins that were found to be enriched with LeishIF4E-5 are given in Supplemental Table 1, sheet 2. These were analyzed for by the GO term enrichment tool based on cellular component. All GO terms were enriched by at least three fold as compared to the gene sets encoded in the genome, with a P value < 0.01.

**Table S3.** **Mass spectrometry analysis of peptides found in the different bands obtained from eluted LeishIF4G-1 and LeishIF4G-2 fractions.** Transgenic cell line expressing LeishIF4G-1 and LeishIF4G-2 were subjected to pull-down analysis over streptavidin-Sepharose beads. The eluted fractions were resolved over 10% SDS-PAGE and subjected to western analysis using specific antibodies against the SBP tag. Corresponding bands that were identified were excised from a parallel gel and subjected to LC-MS/MS analysis for identification of LeishIF4G-1 and LeishIF4G-2 specific peptides. Raw mass spectrometric data were analyzed and quantified using the MaxQuant software and the peptide data were searched against the *L. amazonensis* LeishIF4G-1 and LeishIF4G-2 proteins listed in TriTrypDB.
