# Supplementary material for: LeishIF4E-5 Is a Promastigote-Specific Cap-Binding Protein in Leishmania
Source: Int J Mol Sci. 2021 Apr 12;22(8):3979. doi: 10.3390/ijms22083979 (PMC8069130; doi:10.3390/ijms22083979)
Supplement: Supplementary file 1 [file ijms-22-03979-s001.zip › supplemental figures with legends.pdf]

Supplementary Materials

4Es	% Similarity to *L. amazonensis* 4E5
M. musculus eIF4E	32.6
L. amazonensis 4E1	32.4
L. amazonensis 4E2	27.3
L. amazonensis 4E3	22.6
L. amazonensis 4E4	21.1
L. amazonensis 4E6	43.2

Figure S1: Sequence similarity of *L. amazonensis* LeishIF4E-5 with other paralogs- The table shows percent similarities between the LeishIF4E-5 and different *Leishmania* LeishIF4Es, and the *Mus Musculus* eIF4E.

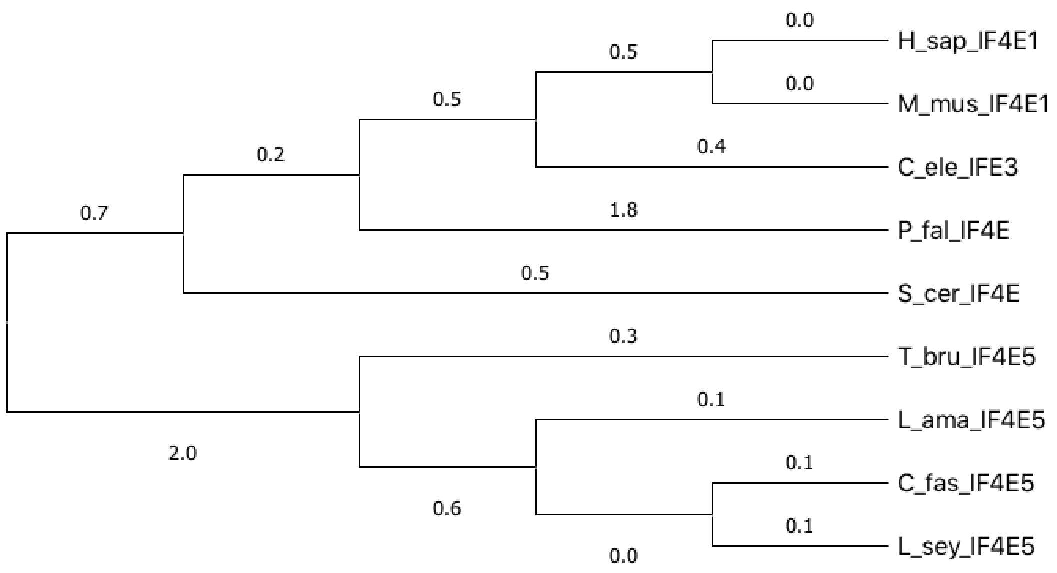

Figure S2: A phylogenetic tree that includes LeishIF4E-5 and its trypanosomatid orthologs, with different eIF4E-1 orthologs. Phylogenetic analysis of eIF4E from *H. sapiens*, *M. musculus*, *S. cerevisiae*, *C. elegans*, *L. amazonensis*, *T. brucei*, *C. fasciculata*, *L. seymouri* and *P. falciparum* by the Maximum Likelihood approach. Initial tree for the heuristic search was obtained by applying the Neighbor-join and BioNJ algorithm to a matrix of pairwise distances estimated using Jones-Taylor-Thorton model. The tree is drawn to scale, with branch lengths measured in the number of substitutions per site (the number of changes or 'substitutions' divided by the length of the sequence).

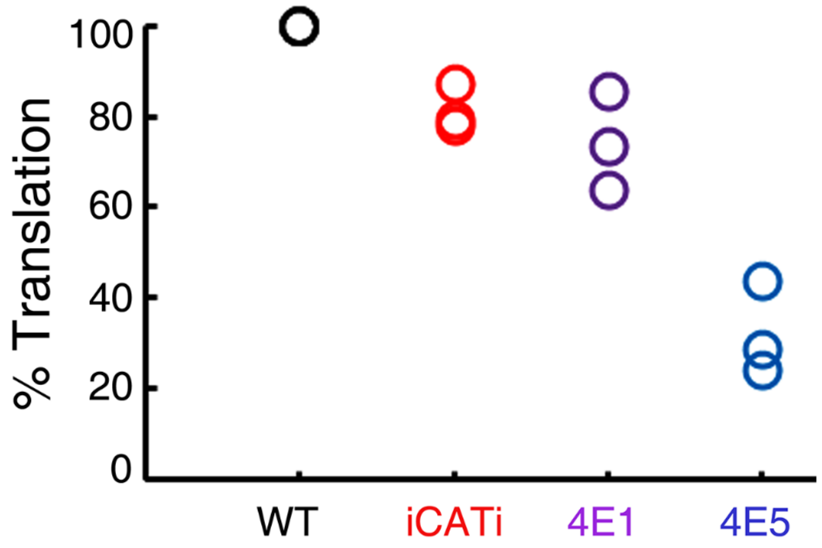

Figure S3. Densitometry analysis of global translation in transgenic cell lines. Global translation was monitored under normal conditions, and the analysis was performed on each lane of the western blot shown in **Figure 2B.** Each lane was fully quantified using the Multi-Gauge software version 2.0. The values were normalized to the protein loads and presented as dot plots.

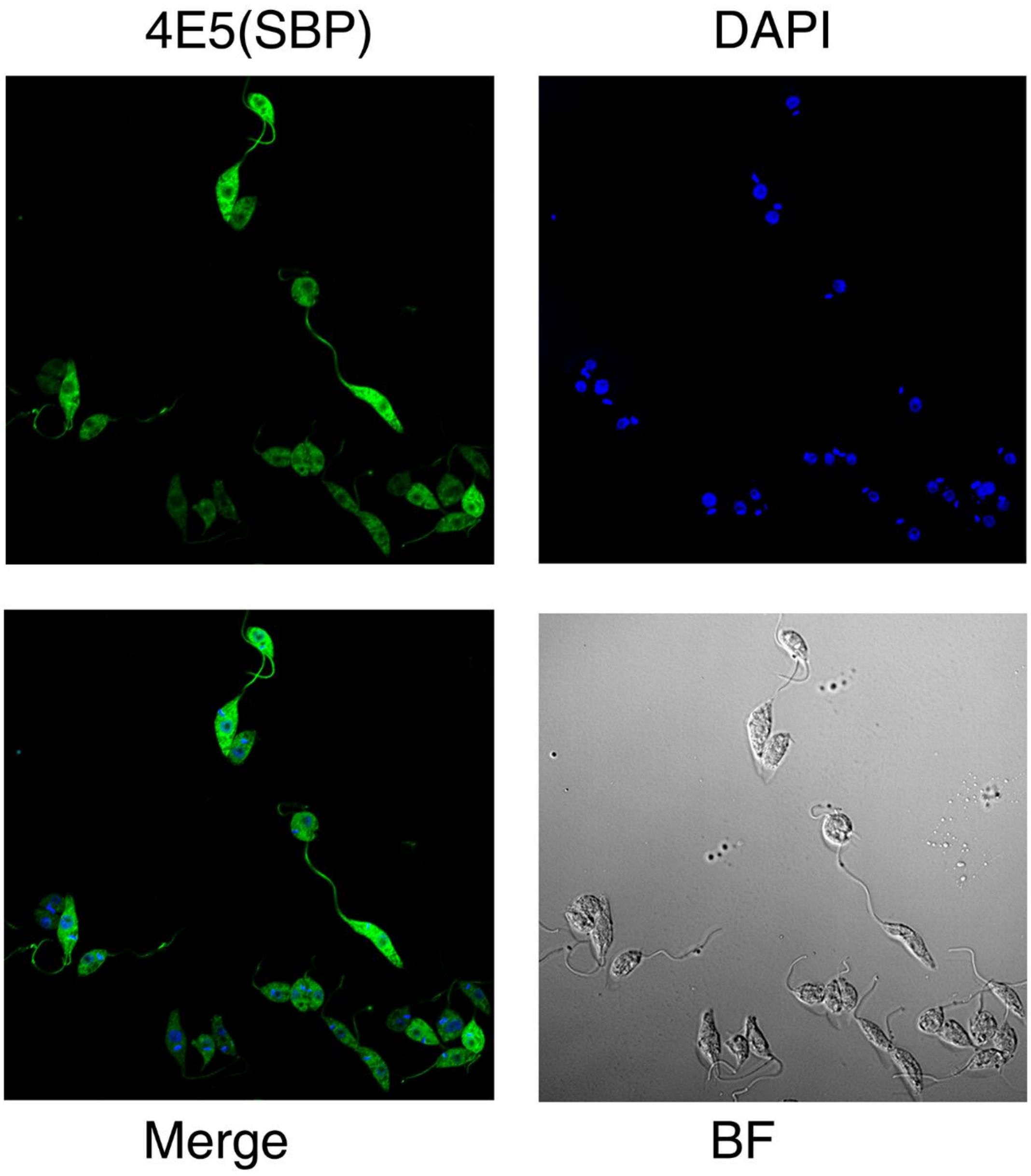

Figure S4. A field view showing localization of SBP-tagged LeishIF4E-5. Transgenic *L. amazonensis* promastigotes expressing SBP-LeishIF4E-5 were fixed, permeabilized and processed for confocal microscopy. SBP-LeishIF4E-5 was detected using a specific antibody against the SBP tag and a secondary DyLight-labeled antibody (488 nm; green). Nuclear and kinetoplast DNA were stained using DAPI (blue) and a bright field (BF) picture of the cells is also shown. The confocal analysis was repeated three times.

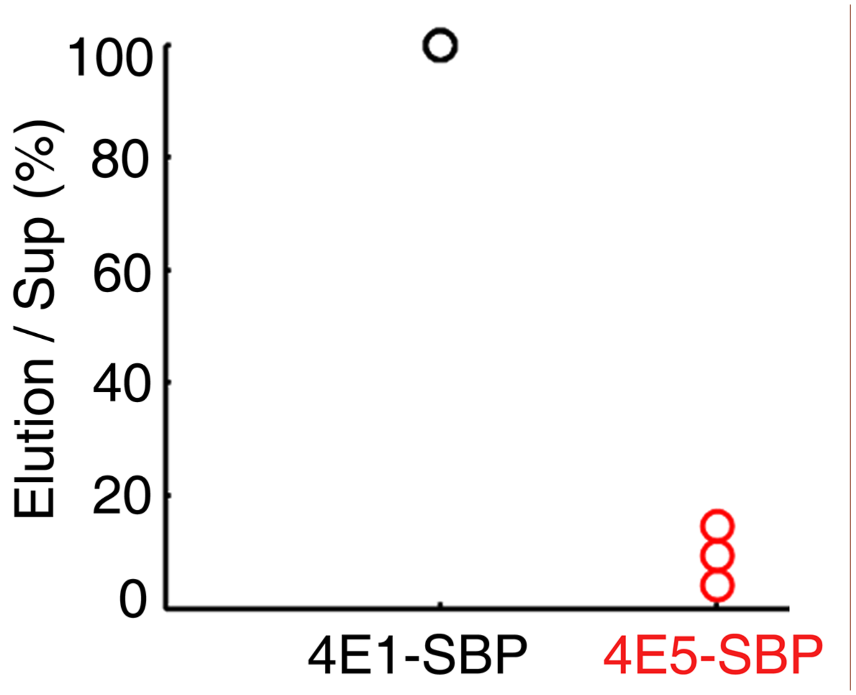

Figure S5. m^{7}GTP binding of transgenic SBP-LeishIF4E-5 as compared to SBP-LeishIF4E-1. The cap-binding activity was evaluated by the Elution/Supernatant ratios. The dot plots represent the ratio of eluted proteins to their corresponding total loads. The ratio of eluted proteins in SBP-LeishIF4E-1 (black) and SBP-LeishIF4E-5 (red) were quantified using the Multi Gauge software version 2.0. Densitometry analysis was performed on three independent experiments.

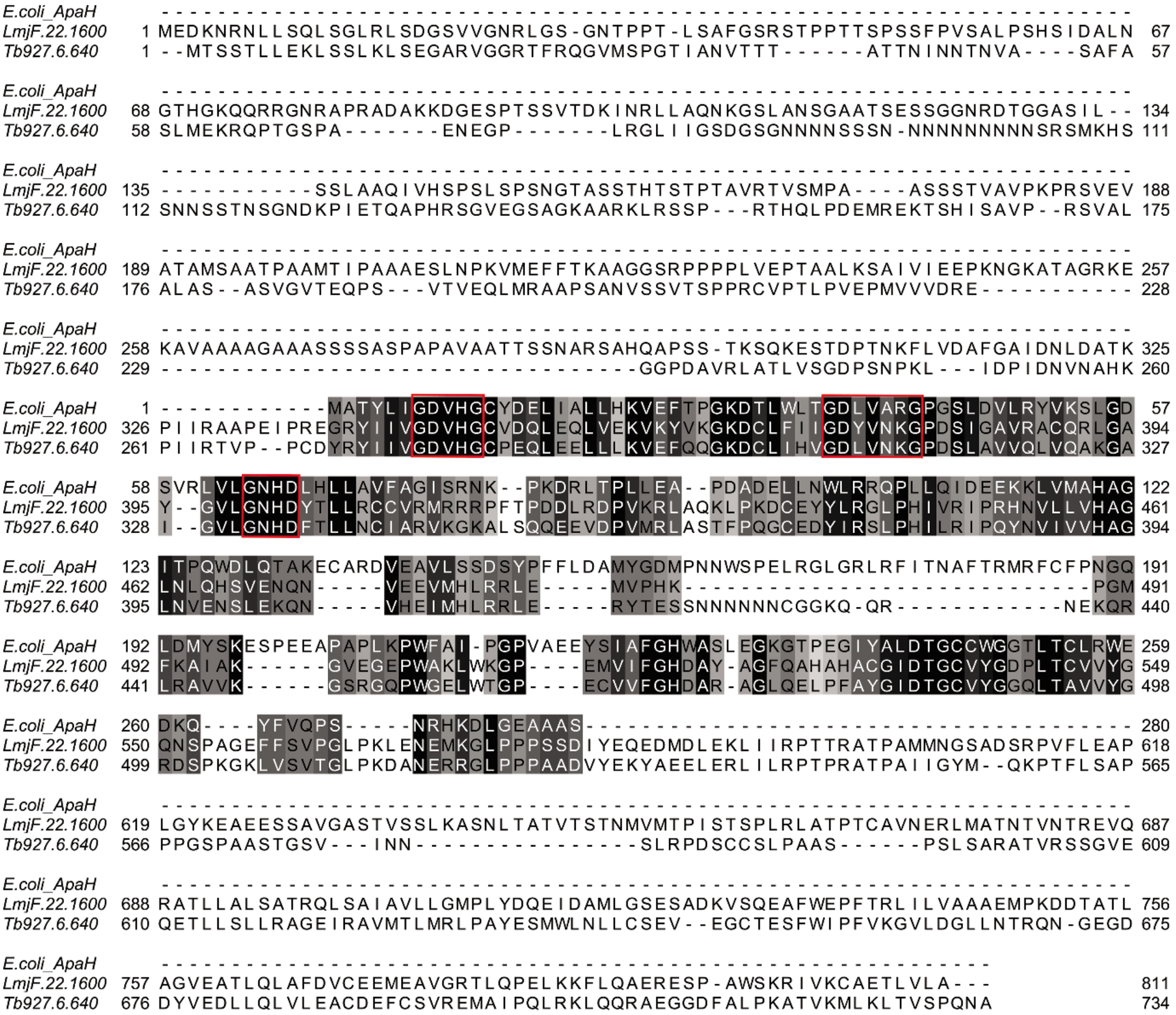

Figure S6. Sequence alignment of the putative LeishApaH-like phosphatase with TbALPH1 and the bacterial ApaH. Alignment of the signature motifs of TbALPH1 (Tb927.6.640, an ApaH-like phosphatase), with the ApaH of *E. coli* and the putative *L. major* ortholog LmjF.22.1600. The three catalytic signature motifs of the protein phosphatases (GDXHG, GDXXDRG and GNHE/D) are highlighted in red.

```
>LeishIF4G1 1015 amino acids
MLMETQISRSLIERERRTESVFRPPKFVKCVPPHKKTLTATARPWYPTSAEVEKKPLTPRLILAPPTALGQSPDVKPKSAPVLAPLTDLS
MQSLQSSLFSPLSTPVHTFFHTITEVATAARVYPVEMFMSVRKDASHTPHGVLRWCVQQYLEVPTLKVPTHLQLSMSEVLNSEDGISRGA
LGSGLKGRTRAKPVRQVHHKVMSVLSRITPQKFTELLQELMQLPLRQADESELHEVVKVFFDKAVQEPEYSELYARLFSELCEMKDGERE
LEAELRDRLFCNRINRELLHTCDEEFHRPIELTADEKVDRTTGKPYSEEEVDMKRTRLKNRLVGNIKFLGELFKINLVTERVVENMFKLL
VGDFDPSNPVEREDYIFEVFSTLIRTVGPILKERRPLMLARYLGVAKAVENSHPRPRIRFLMMDLSDLNKKQGWVSSERVMREADSCDDL
QRSSSMKHVDSVTSTSFLQSSIMTPSEDNTMQRCDFALSRPASVAFRDNSGFGASITLSPSTPSNSPTLSRTYVPPKPASLSPSHLRPSY
NAPMQGSLPERRSPASAYIRPSIDLPASHSLSNAPPHSSLPRSRTSMVGATEVVNIQEVTQQLIQNFNDPREEATVLNGISAMSLKNKVL
CLTWWLRQVTTDTSRFEERQRITELLSSILTSCPSFQPSDLISAIIEWIRFDIEKAEYHQCPRMFENMGHMIQRCYAADVSTLPNINEVQ
NLLHLGLFNVMLHDLTTSNGTDQMVTLVKSAFSIGVSLLNTIADRKSEAATLRVALQCRFRLLPYFFSVSNLSETGIPCTTPQCRTPTTP
NTRNIPQMESISSVGHYDPLSQCMLFHKVADDAEFVVLRRARADASERTAAWKEALVSLALSETGNGSTISQLVQMAKVIGALLACSTVS
VNGANLLSATEVDELVGEMLKRCSGPLFQAMAAMEVIMHHTLSVSGSNPRKSIRVRQLRLMYSRWCTLNIFDTTVVMNLLQSLHAPENGS
SVYAVYHNALVDAELPWATVLTYLS
>LeishIF4G2 1453 amino acids
MEHSCRGVRSPARRCSSVPRNLEGSRSTPSSFYAFPSTLNDILFSPRQRLTKYADGMQALPYASSAPTIEEAEVAAAGPLADARAEDAAT
AAVTEVVQSSSLEAAEKQAAKEEAVTVAPAEQEAANKPLEEDSAAAVAVAPAETESAATAQIGANAPDDTAAPTAADEAESMAKEEEGGR
TDASLDYVADEAPSCSAVVATADEAEKDTATKDDAAAGEVAGRADAKTVPEEAAAEQEEGSETAEEAQRSSDAAERVGAEERETAAVLQD
HGRDEVTAAAAEPSAAPEDESPAEPPQLRTTCDIVAFDAAQIAAWMAEYRHETAHVPPGVQRFVHEFNERAAKSAPLRMGGTSPTPSAIG
TAAKKGKKGSLSLSSVGSSKTGIGALADLPCRDGGGFGSPAALRIGTSVAVKRSNALEALGDVWEGRQEAPISLAQGSLRRRGLFGEVEK
KMIHHMVVAVLNKVTTDPVKFREVKNELLRLPIPEANTEQMTKIVDAFFTKAVREQHFSHSYADLIAALCKVPQGQHIVGDKTQSLEYRL
RMALLKRCQAEFLQSIRAEETDASSPTAMATAVDDTSSPLTSAIEGAQAIGGETEKERRDRMCGNVRFVCELFLRDIVAASVISVIFRIC
CLGSEFGEFAVPPSYTPTESQVDEIITVVKTAKERYFVQSAEGRRMLPQLLSQLEYWVKHYSISRCRFVLMSTVDDLRAMLPKEPASPMM
SSAPSVAAFVTPQLSHISPTQSPPPCDVSEQGVFSAVSPAPLSAAAALSAVTAADASGQLSMSSTMVCGQPLPAPRSKQVGSVSLSDSAV
SINGMATAESPVTGAITRPIATLTSRSTLQHVRPEAIAKLMAAFSSGQCSADEVAVQLFDTYGNVLPALGAWMDRCLSVVKEEKTRKQTG
AVLVACVEQLTAHTNATSEEVAETVAMCREQLHEVAMEALQRAIEGKLYEDLHVFQFWAQLVLSDHARVVYDEELLNEGLELLVYNAPPA
VRSYLVEVGKYMTHVLVAPEKLPWQASTEAQSFVRYRPLLVLHSLVMPGGPSEAQALLDNIVGFSDVRQQSLELRFYHAFRTGSPSREVI
FEQLRTSPRTTSRDSTLAAEVLSALLIAELCSNGDALVEDNMDLLQITVDGVTRAEREMAIVTEVYEILRYTPRPMTMSAAARVLRKFVC
MHILSDETMERADQFYEAECDGAIEKPTQEGGRALAAPPSADYPRIMDRRGTDDVLLSGSISMRSVNGAVATNSNGNGNNGGGVGGGTGS
VDGLQSSNASSFGEFRPRDSHPPRRDADSGHTSTRSGSCHRGGATRATAPSRRHAAARTTASGCTATPTRTDGAAKQRAETSGTPGQGAA
RAANQHSSSSPQGRCRRHPTTLVVTTVAATAVGVATTVSTAARAPGEAAVTLTVAVAVVVVAAATAPHFIGGAVAVARLSNGADLSRCTV
VLAGNVRCAKSAR
>LeishIF4G3 635 amino acids
MQFTVEQIRSVRNNYLEPPYPGFSLDEVIRRRRLTQTKLVRGENAWVAKGTAQTTEEWVQRLLHGTLNKLTEENKEIMIDKLLTKELFAT
EDIMNMVVNIIFKKALDEPENSKLYAGVCHSLALYEANVLRDGHKGERPQSQLRDAIISTVQHEFRTLSKELSKMEDKSEEELEYERSNV
MRRKRSNMRFIGELYLCTALTHSTMFTVMDLTMRCSDKTGFPSSENIELLAALLDTIGDRLDKSHKKTLDRYFTSLENFNKKSDCPYPPR
IRFKIMDLLDLRKRGWGSKPKPVKTALPPSHSKDKKYATAPPPKKGGRDQGSPATASKSWRDATTTKTGTAASPAPSVDKGLNGRGTSSA
APAAKTAALSSSAAATAAATAPVGASFRDEASPQAPLVKFELRVASMFQEWVADRTNDVILHWVDQFNTCDRFFESESELCVVVAQEVIH
SACTTTRKDAQREAFSFLVVGLYMIDTEVFDGFASALASAIEDGLLEDVPKFGERFMSMLRLTSTEQETRADVYFDAANVLRLTYNRLES
PDDSAVDTLMSFWDRVPLPSTDEENMIRMDLDVVYSLCNPEGAQDGLEKLLSRIIHSMLQMQLMDEEVLDEFLCLDVEGGLCAKVIADYK
ERFPK
```

Figure S7A. Various *Leishmania amazonensis* eIF4G Sequences showing the presence or absence of the $Y_{XXXX}L\phi$ motif. The $Y_{XXXX}L\phi$ motif is required for binding to theLeishIF4E protein. Y refers to Tyrosine, X refers to any amino acid and L refers to Leucine. The ϕ residue represents a hydrophobic residue, but it is not conserved in *Leishmania*. The $Y_{XXXX}L$ is present in LeishIF4G-2 and LeishIF4G-3 while it is absent in LeishIF4G-1.

```
>Trypanosoma brucei eIF4G1 1118 amino acids
MNCSAPGLYSLVTNEVWKPSVFRPPTLQKRSRPVAKTTLTPMAQPWYPSNANGGCDSTSSGPQSCQKDGPRLLPPPALGQSPTFGSDIEK
EFPTVLCSSSVVAPTATCATRRASAFTKAAMEARTYPLDMFMSVSKDAAHTPYGVLCWAVKQYVTCSYLKVPTHLQLSLRDFGGRPAEVD
ARGLMGTCLKVKSRAKPVRLTHHKVLSILSRLTEAKYDTLLSELKLLPLRQVEDDELSEICKIVFEKAVSEPSYTSLYARLAQDVCKEKK
EERDSSVWNEGFQRRFRRTLVKLCEVQFHQPLQLSGDDVVDRTTGTPLDEEEVEMKRSRLKHRLVGNIRFVAELFKVGVLSGRVVTEIVQ
ILVSDYNPDAPTAKEEHVFELFTTLLRLTAAQLKDCEVNLLIRSLGIAKTIELSHPKPRVRFLMMDLGDLNRKNGWVEQEQIQSHVKEPK
QLKKVPGPAPAAPASPTTSTPTAKRAEKLGSRLRDMSSRCCADAEVSLRKENGNGTQTPGSTSYGNKVADGCADGGNNKNNNGGVSNAVK
TNSNGKHTSRTGIENRSGSDETAKVKPSNGAAVPSSAKQSTNASRRVSHNPYCKLSPANILDLKNGDAKAASGNSNIPYTESPLSPAASS
TDLSLSSSSSGHFHNNQGNGGTAMHSGLCSPFLERDKMVNERKMNIEKITMQLMALFRTGDEETTATILHTMGLKNMVMCLTWWLRLATT
ASGNSDNAQCYGDYSKVAALLSALLSIRNDAYATSTVFSTVLEWLRFDMEKHEYESFPGMFEKIAQMIRQCHLPITDSLPNVEVVREIMH
GGMFNVLLRELIINGGITAPVSDLVKSCHPASAEIICHLHDPADDSQMLFVAAQNRFHFLSYLLCVSSMTRIDAQSGFHLRTPLLPPRCN
NSQAEWTPLVECLALPSVASDPESLLFVKMWDHATNGDAASLWRDEAIRLALETPAGQNSTTSSLISVMRVVGVLLMGLVTKCMTVGACG
TNNGSSGGNASGAHALAETADVHYVVSGILKQHPAPLYQAATVVELVMHHTRALSGTSECPTFDNARLKALKNEFDDWCEAGLVDRPAVL
ELLRVVDATKDNFNLYPAYHNCVADVPWHVAIRSLGGN
>Trypanosoma brucei eIF4G2 998 amino acids
MDSPEACRVQRFRRTSSSRDSFNIPTYVRTVPSALVEALTAPREKFERYHKSSLTDGSKGPRETSQKVNQGTQTNPIASDSKAAQFSGSI
SDCEAVAPLGSKAVTAPPRDATTPEGEELPMRTTESSAKPSTNITEERSEVEEAKTNDNIPRCIPYDPEGTVLAMQRLRDVKHRCPAKIA
NCLQKINKEIAEKLTNAGEALSASPSTSLSSVTVAGPDGRQRTGPPAPLAALPEPWEDRLDAPIAVSNTLGRKRGLFGDVERKELHHHVI
SILNRVAGDATKYREVKNELLRLPIPEASDEMLKNIVNVFFMKAVREQHFANLYADLVVELCKVPEGQRIVGNKSQSLEFRMRQQLLSRC
QEEFQRLEEKNIETANQGGEDRNTQSADESFKERRDRACGIVAFVGQLFLRHIVTEKVIMQILTTTTAGHYGREGLVMPPPYTPSEGQMD
ELVKLLGIVDSAFFATPLGSSMLVVFTDIMVHWSQHHPVLRIRLLLLSVVERLQKKIGEQNAAAQQQRQQQQQQPPGQTRQSAFEGKRPP
NSVGDSQRPVSPSSGGNSPLSVSGVPAAVSVAGVEVAARPIQPPPAARNSLIPAPRSAASRQSSIVVTTPLTSTVSTSFAVKQVATTETV
ATYMHNVTRCDCTVDMVVDEIANAFENVISVVVVWTERCLTVVKTAKQRVLFGPFLCSVERQCGGTLKEKLRQVAMDAFRNAVRQKLYED
LAIFKYWTEMICSDTAREVLDETLLNEGLDCVMSFDRSAVRIYLRDVAMQLQQRSSGPQREPSLEKNDFIRYRPLQVIHANTAESGVEPI
LALIDYPDVRKQSIEIDLYCTIVTDNPPKNVLFDSLASSPCLESPLAAAEVFSALLHAWLKSGFANSIQDHVDICLLAVNNRDRATREIL
LLMELYSVLKNVVPPSTRSIEMGEIALKVLKKGLVTEETRVRAYNYLEPLERNPHHFIGIPPGSIARSEFESSKPVISTLKERKSHSGNG
SSGAVRRV
>Trypanosoma brucei eIF4G3 622 amino acids
MHVYTIDQILELRSLYPEPPYPGFSLEEACRRKKQTQTKLVRGPNAWAARGSAKTTAEWVERLVYGSLNKLSAANFNEIVSQLQTNTIFS
SDEMLKKTVSIIFNKALGEPENSNVYAGLCYKLAEYEVSLNVVQKQKEGKRLSKLRNAVVGIAQTEFQNRRNVPSSEGLSEEEVEQQRSS
FMRRKVANMRFIGELFLHKVLSHSTMMDIINIIMQPEKGGYPASEDLEFLTVLFTIVGKSLDSIAELRPKLDAYFKVLEGLKDQKIYPPR
ICFKMLDLIELRRDRNWESRETVMPKTSAATQKEPPRASDRAVRSSTTPNSSNASSSGSKKGKGAGPCDSALGQRAAASTVGKDAREGLK
SRAWRDVVKSEIVACDDHEMTVTESVAFEERVRSLFQEWLSGYRTGCLPNWQEEFRDCGARPIPDMDLPTAVAAQVVREACMTTRKDAQC
EASRLMVIGLFLEDDQVFNGFAAALSSAIEEGILEDVPKFSERFANMLRFTSGDDVKTDVYYDAVRVLCTAYGMLRDPDEMSLSTLMEFW
GKIPPPEKKEDAIFSLPVVQSLVTMTKLGRAQITGHIISSLHANGLVDDATLHEWLGTPGGEVDAEVKDAFRKATVGKGTLT
```

Figure S7B. *T. brucei* eIF4G sequences showing the presence of the $Y_{XXXX}L$ motif. The $Y_{XXXX}L\phi$ motif is required for binding to eIF4E proteins. Y refers to Tyrosine, X refers to any amino acid and L refers to Leucine. The ϕ residue represents a hydrophobic residue, but it is not conserved in trypanosomatids.

A

4Gs	% Similarity to *L. amazonensis* 4G1
M. musculus eIF4G1	21.5
L. amazonensis 4G2	22.9
L. amazonensis 4G3	27.4
L. amazonensis 4G4	24.7
L. amazonensis 4G5	30.4

B

4Gs	% Similarity to *L. amazonensis* 4G2
M. musculus eIF4G1	26.1
L. amazonensis 4G1	22.9
L. amazonensis 4G3	18.1
L. amazonensis 4G4	18.6
L. amazonensis 4G5	20.1

Figure S8. Sequence similarity of *L. amazonensis* LeishIF4G-1 and LeishIF4G-2 with different eIF4G candidates (4Gs). (A) The table shows the percent similarities between LeishIF4G-1 and different *Leishmania* LeishIF4Gs, as well as with the *Mus Musculus* eIF4G-1 **(B)** The table shows percent similarities between LeishIF4G2 and different *Leishmania* LeishIF4Gs, as well as with the *Mus Musculus* eIF4G-1. Percent similarities were generated by EMBOSS needle (https://www.ebi.ac.uk/Tools/psa/emboss_needle/).

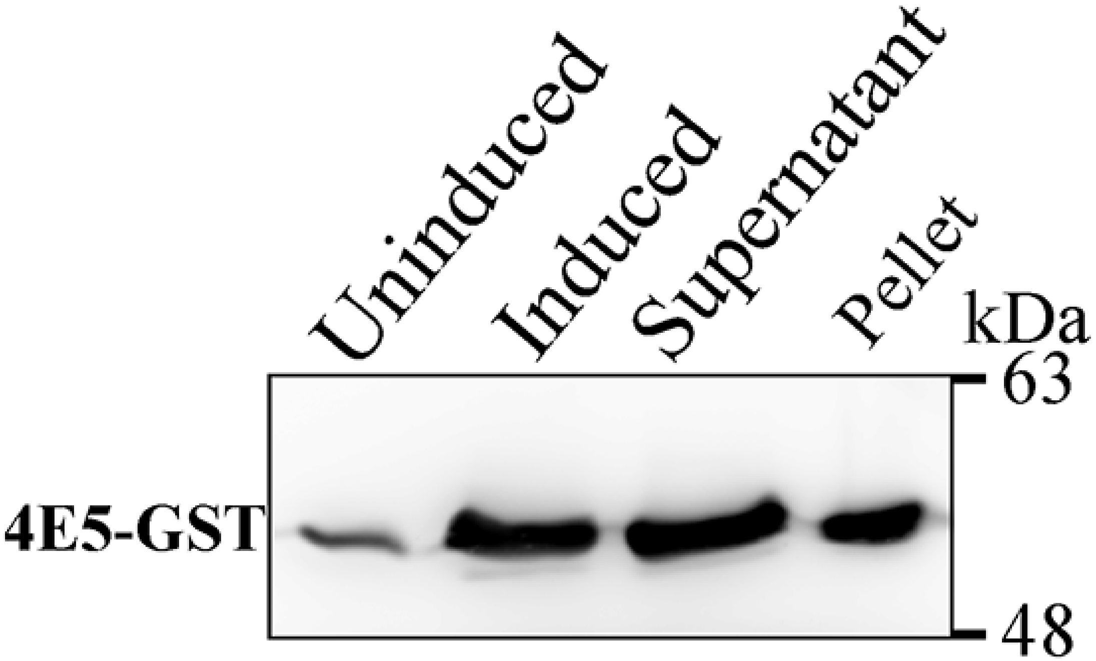

Figure S9. Expression of GST-tagged LeishIF4E-5 in bacteria. Full-length GST-tagged LeishIF4E-5 was expressed in BL-21 cells. Expression was induced by incubation with 0.5 mM IPTG. Cells were disrupted in a French Press, and the supernatant and pellet fractions were separated by centrifugation, to evaluate the solubility of the recombinant protein. Aliquots from the total extracts of uninduced and induced cells along with samples from the supernatant (Supernatant) and pellet fractions were resolved over SDS-PAGE and further subjected to western analysis using anti-GST antibodies.

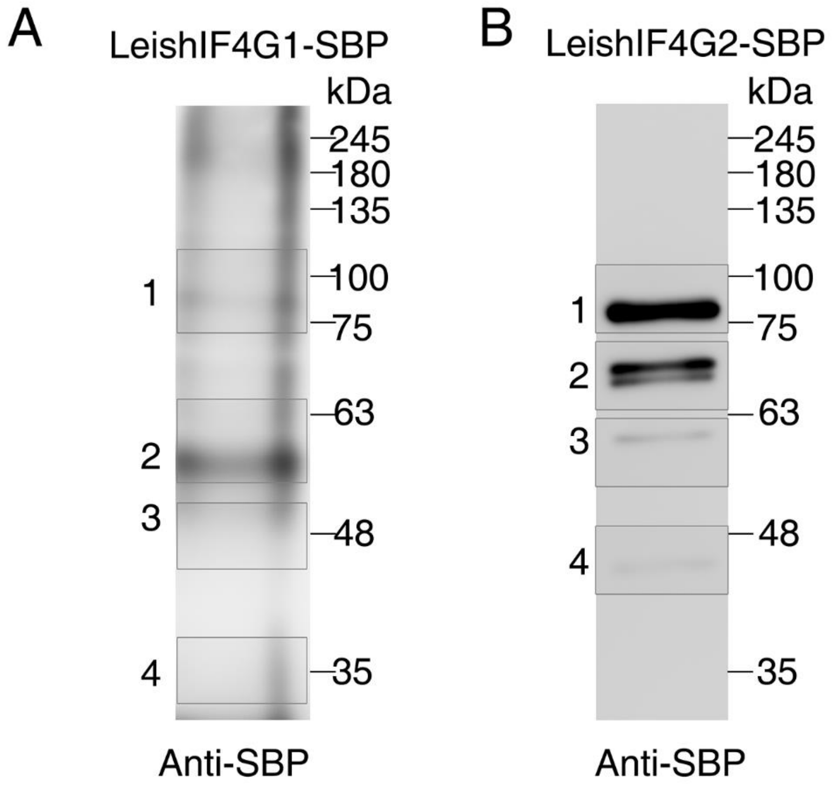

C

Band number	Number of peptides associated with LeishIF4G1	Number of peptides associated with LeishIF4G2
1	24	28
2	06	03
3	00	03
4	00	03

Figure S10. LeishIF4G-1 and LeishIF4G-2 are susceptible to proteolytic cleavage. Lysates of *L. amazonensis* expressing SBP-tagged LeishIF4G-1 **(A)** and LeishIF4G-2 **(B)** were purified over Streptavidin columns. The eluted fractions were separated over 10% SDS-PAGE gel and subjected to western analysis using monoclonal antibodies raised against the SBP tag. The bands that were highlighted by anti-SBP antibodies were excised from a parallel gel and subjected to Mass Spectrometry (MS) analysis, in search of peptides derived from the corresponding LeishIF4G. (**C**) The table shows the number of peptides obtained in the MS analysis of each band. The original MS data are given in Table S3.

Table S1. Manual categorization of proteins enriched in the LeishIF4E-5 interactome. The LeishIF4E-5 associated proteins were enriched by pull-down analysis of transgenic cells exptracts expressing SBP-tagged LeishIF4E-5 over streptavidin-Sepharose beads. The proteomic content was assessed by LC-MS/MS. Proteins were identified using MaxQuant software. Their enrichment as compared to the luciferase control, was determined using the Perseus statistical tool, setting the $\log_2$ fold change ≥1.6, with an adjusted P value (Padj)≤ 0.05 as the threshold (sheet 1). Identified proteins that were enriched with LeishIF4E-5 were categorized into groups according to their function. Raw data are shown in Sheet one, the filtered proteins above the threshold are shown in sheet 2 and their categorzation is given in sheet 3.

Table S2. Identified proteome found enriched with LeishIF4E-5 – classification by GO term enrichment. The LeishIF4E-5 associated proteins were identified as described in the legend of Table 1. The proteins that were found to be enriched with LeishIF4E-5 are given in Supplemental Table 1, sheet 2. These were analyzed for by the GO term enrichment tool based on cellular component. All GO terms were enriched by at least three fold as compared to the gene sets encoded in the genome, with a P value < 0.01.

Table S3. Mass spectrometry analysis of peptides found in the different bands obtained from eluted LeishIF4G-1 and LeishIF4G-2 fractions. Transgenic cell line expressing LeishIF4G-1 and LeishIF4G-2 were subjected to pull-down analysis over streptavidin-Sepharose beads. The eluted fractions were resolved over 10% SDS-PAGE and subjected to western analysis using specific antibodies against the SBP tag. Corresponding bands that were identified were excised from a parallel gel and subjected to LC-MS/MS analysis for identification of LeishIF4G-1 and LeishIF4G-2 specific peptides. Raw mass spectrometric data were analyzed and quantified using the MaxQuant software and the peptide data were searched against the *L. amazonensis* LeishIF4G-1 and LeishIF4G-2 proteins listed in TriTrypDB.
