# Supplementary figures and images for: LeishIF4E-5 Is a Promastigote-Specific Cap-Binding Protein in Leishmania
Source: Int J Mol Sci. 2021 Apr 12;22(8):3979. doi: 10.3390/ijms22083979 (PMC8069130; doi:10.3390/ijms22083979)

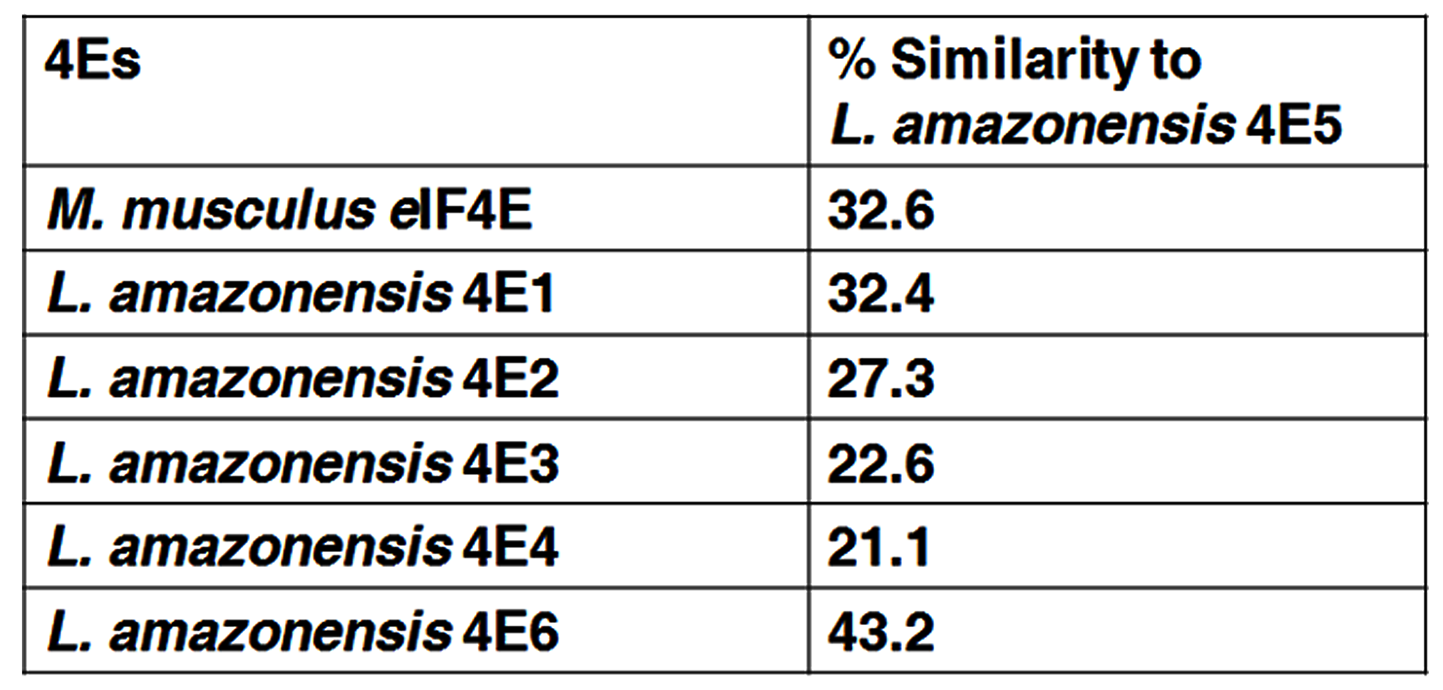

Supplement: Supplementary file 1 [file ijms-22-03979-s001.zip › Fig S1_4Es similarities.tif]

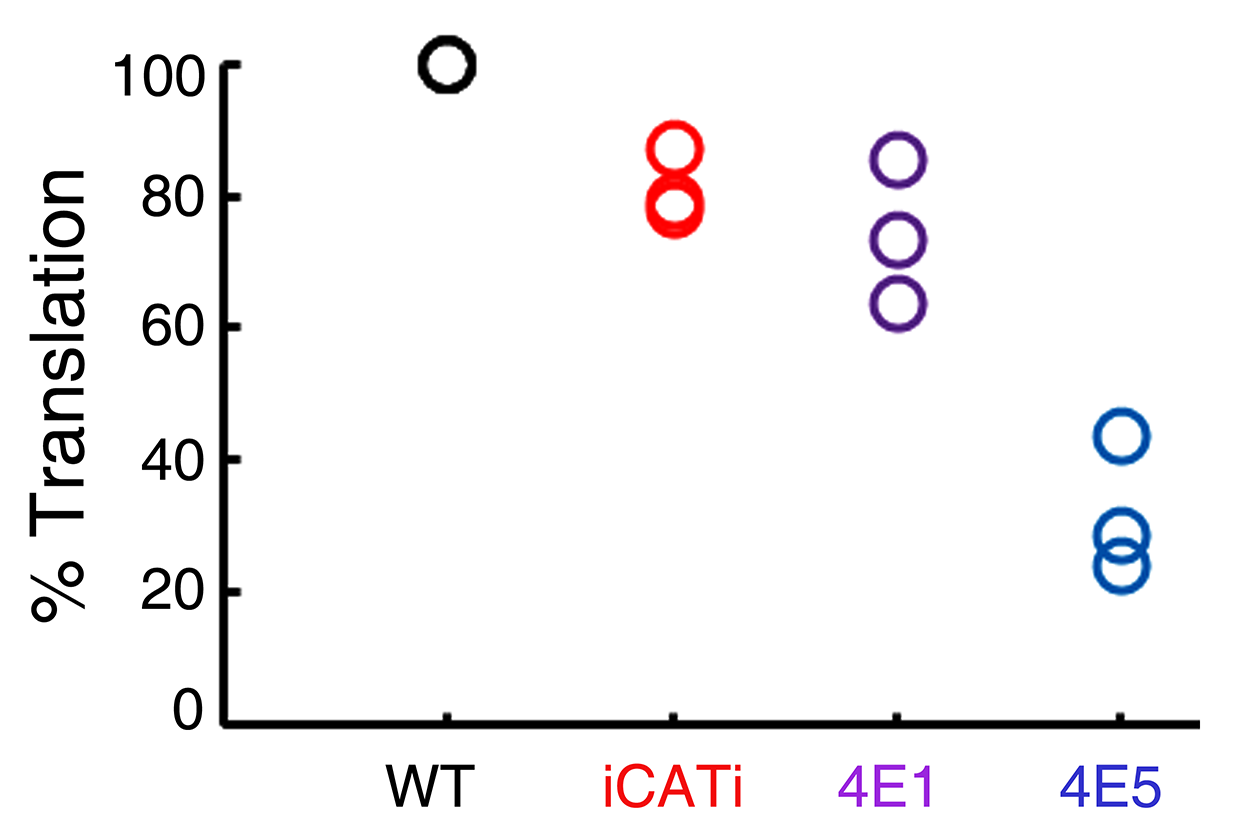

Supplement: Supplementary file 1 [file ijms-22-03979-s001.zip › Fig S2_DensitometryTA.tif]

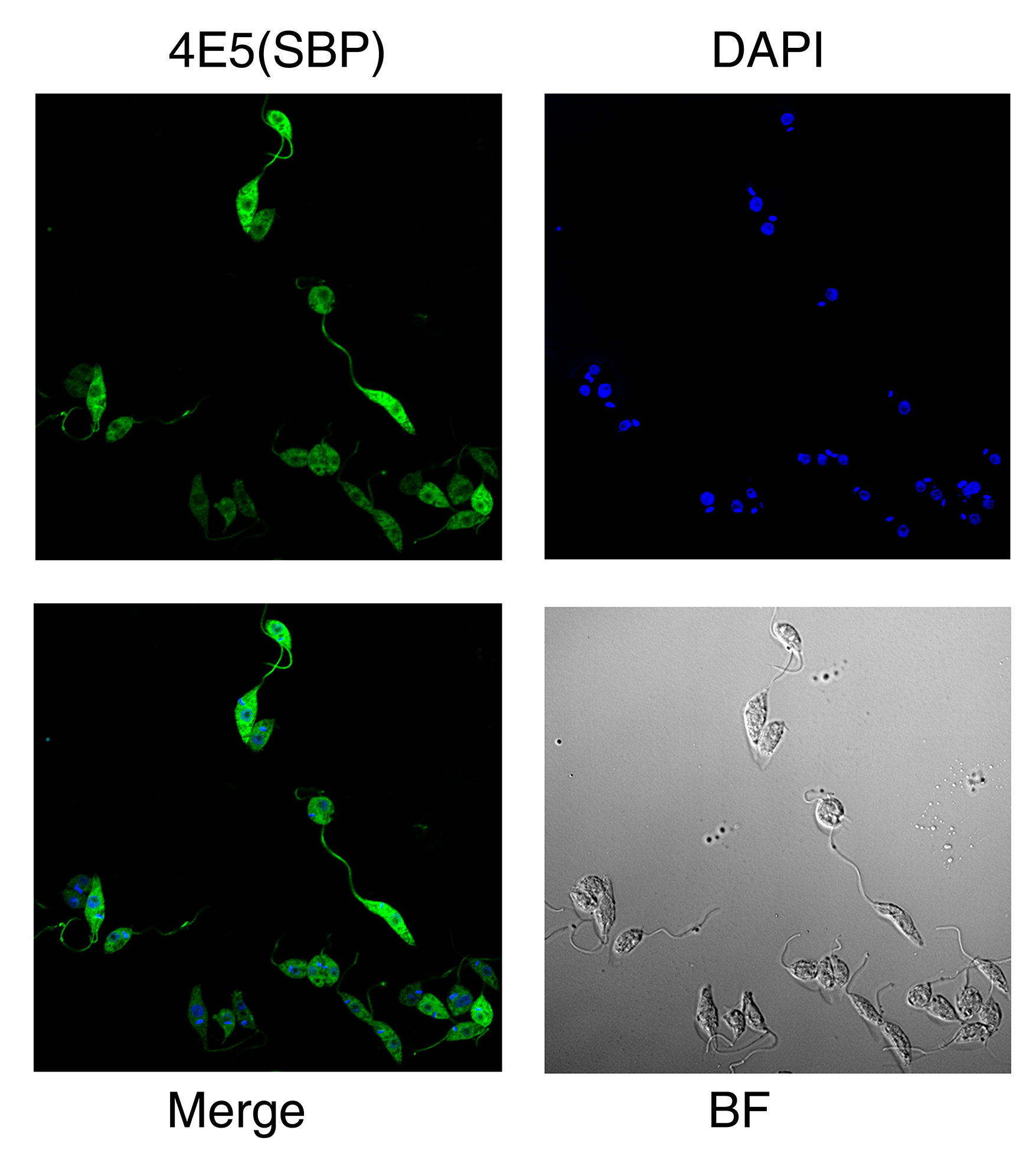

Supplement: Supplementary file 1 [file ijms-22-03979-s001.zip › Fig S3 CONFOCAL_BroadField.tif]

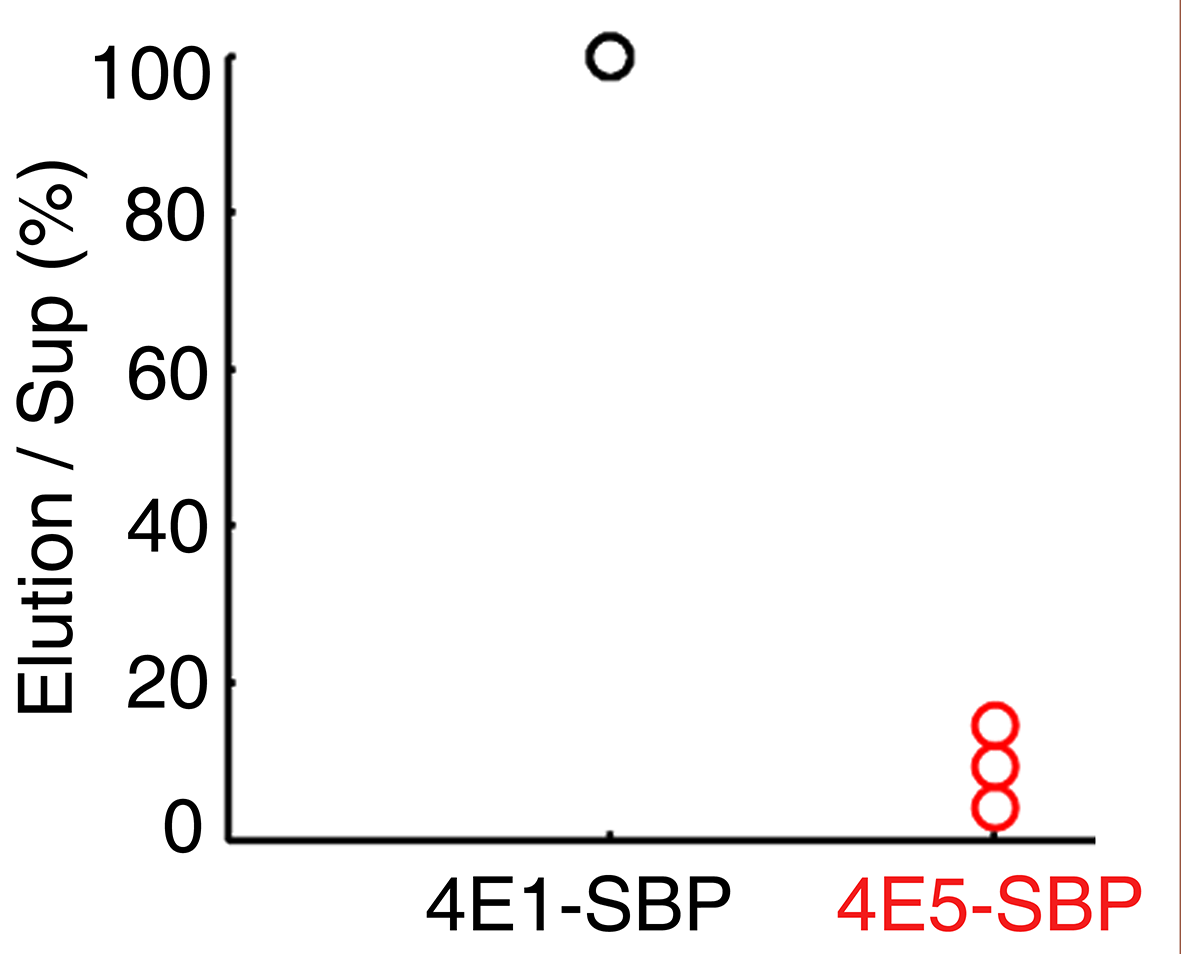

Supplement: Supplementary file 1 [file ijms-22-03979-s001.zip › Fig S4 E_S Cap binding.tif]

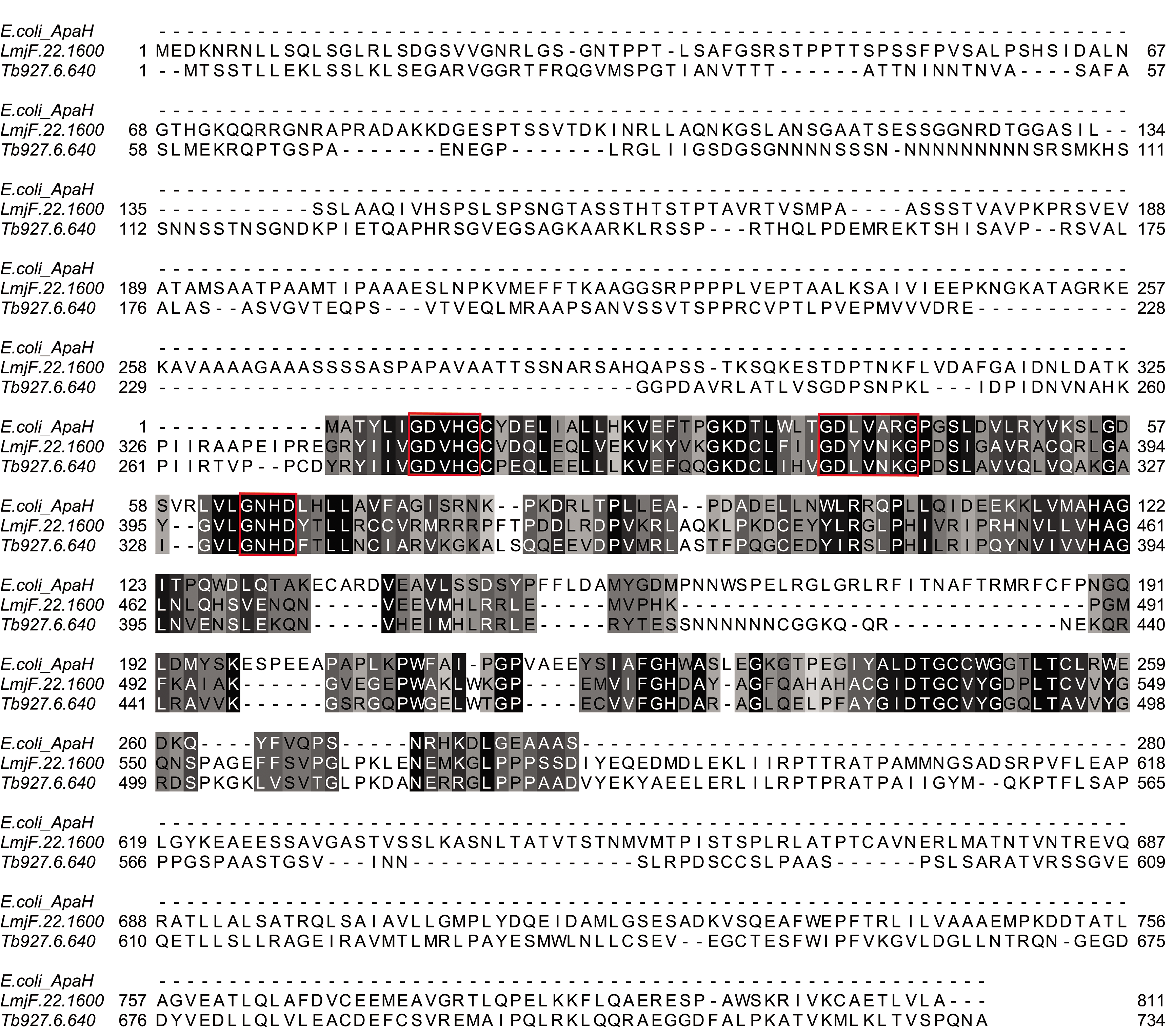

Supplement: Supplementary file 1 [file ijms-22-03979-s001.zip › Fig S5_Apah_Alignment.tif]

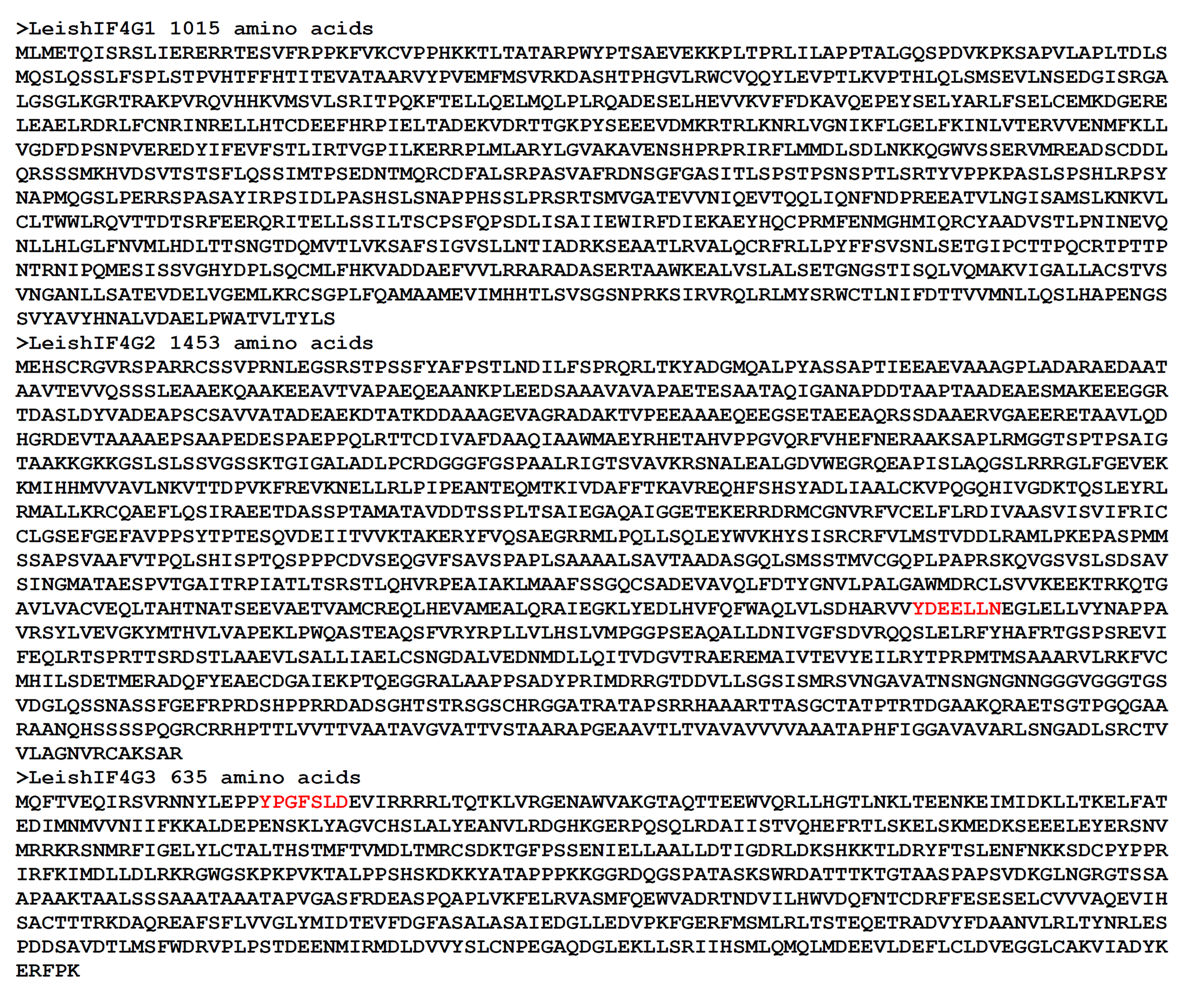

Supplement: Supplementary file 1 [file ijms-22-03979-s001.zip › Fig S6A_LeishIF4G1_2.tif]

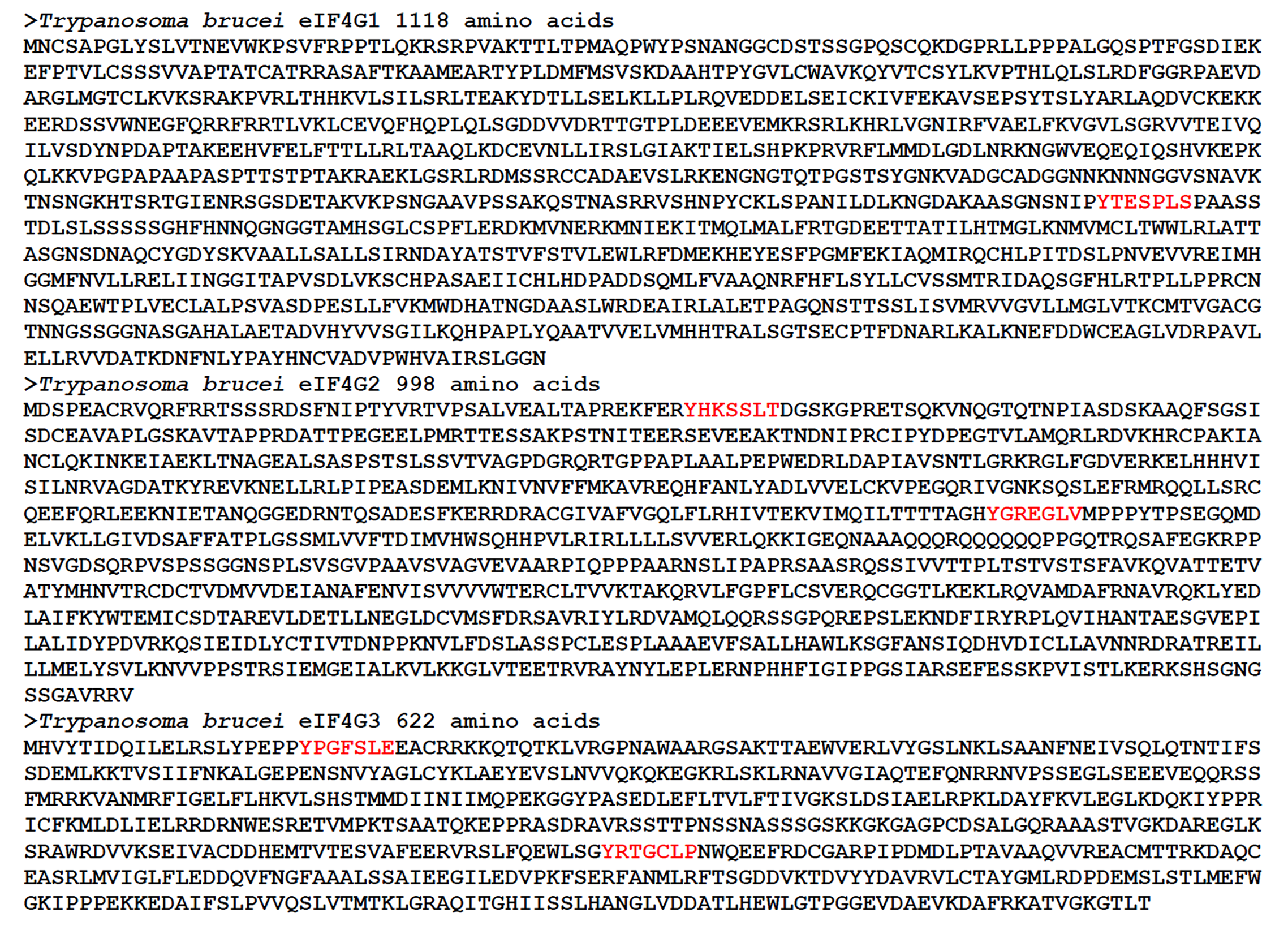

Supplement: Supplementary file 1 [file ijms-22-03979-s001.zip › Fig S6B_Tbrucei 4Gs.tif]

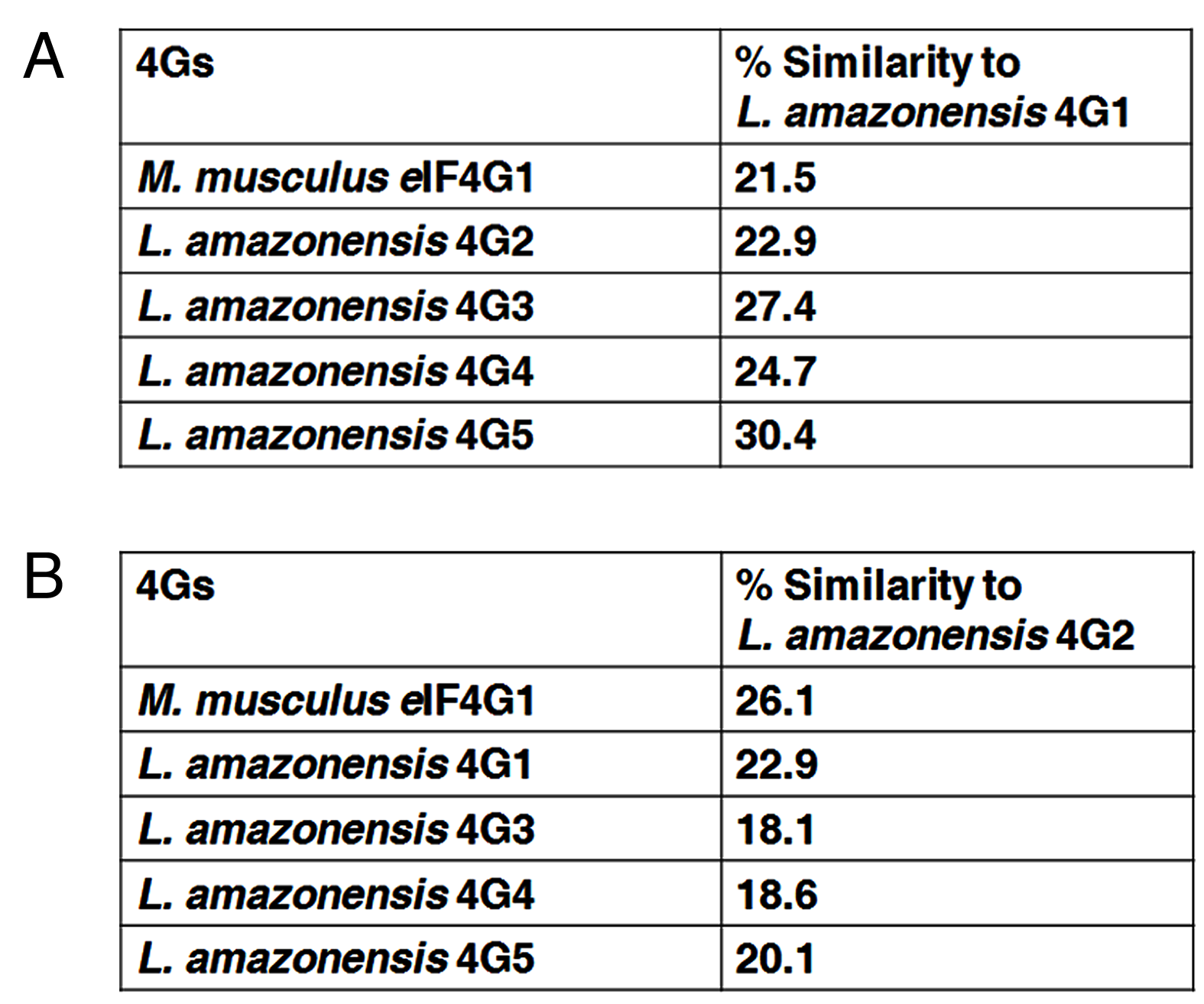

Supplement: Supplementary file 1 [file ijms-22-03979-s001.zip › Fig S7 (1)_4Gs_Similarities.tif]

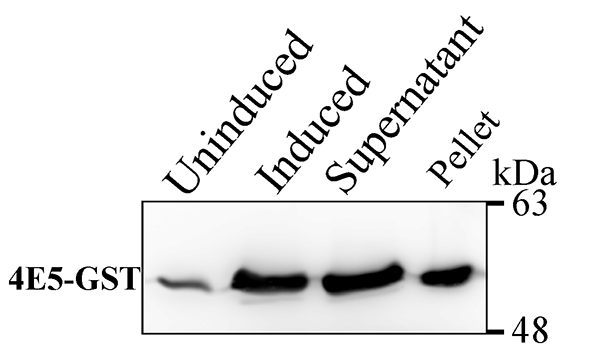

Supplement: Supplementary file 1 [file ijms-22-03979-s001.zip › Fig S8_Expression of recombinant 4E5.tif]

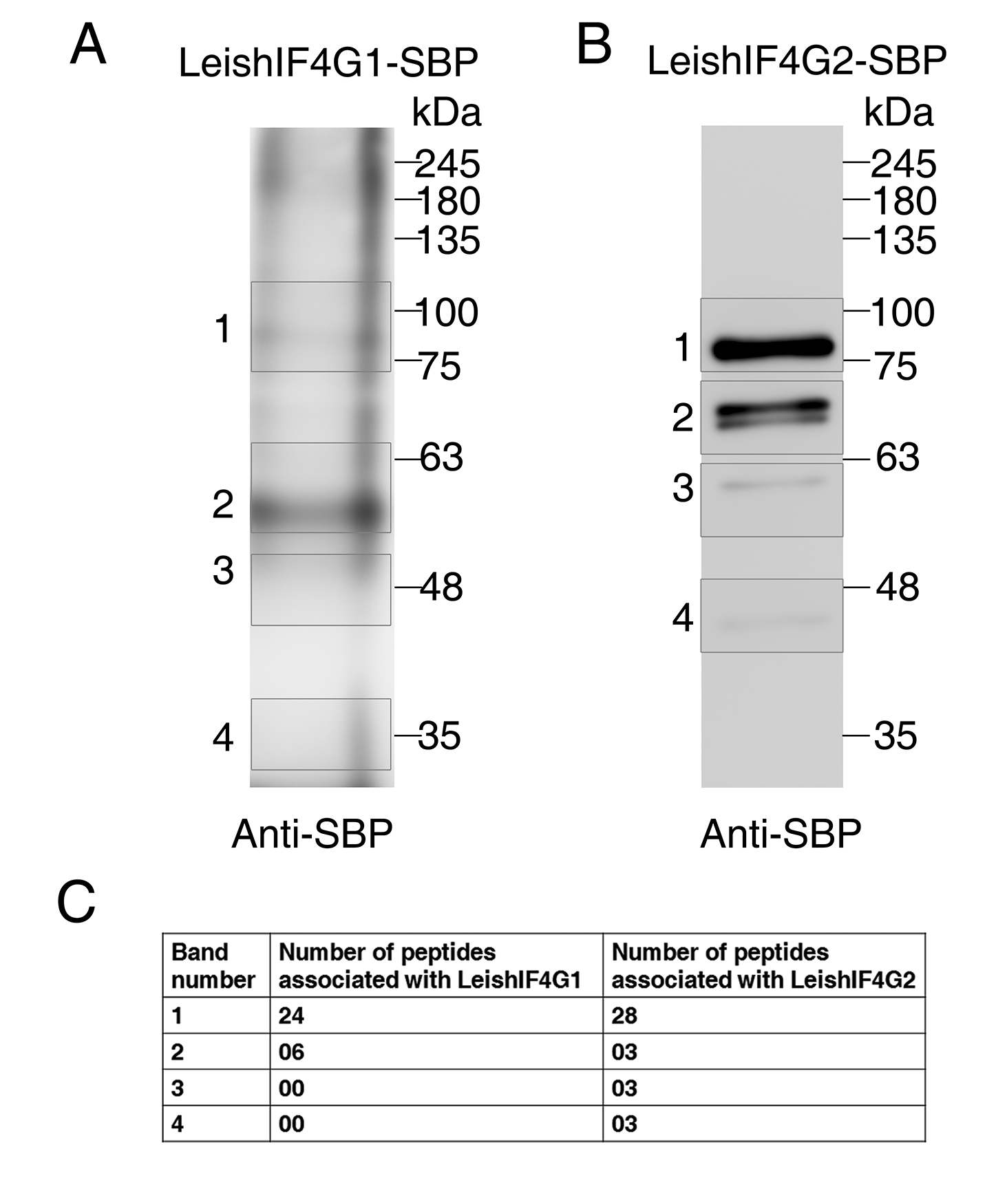

Supplement: Supplementary file 1 [file ijms-22-03979-s001.zip › Fig S9_4G1_4G2 Breakdown products.tif]

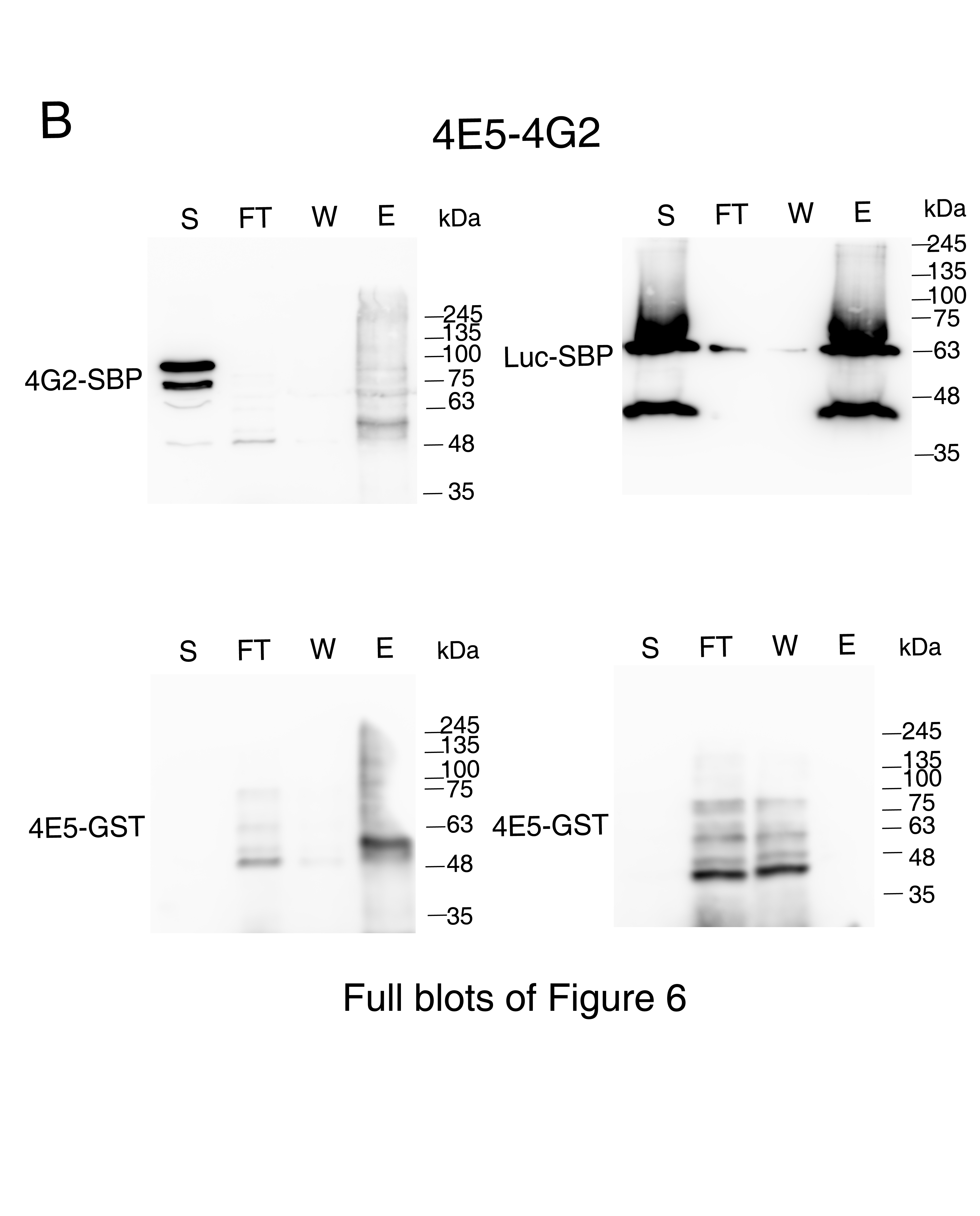

Supplement: Supplementary file 1 [file ijms-22-03979-s001.zip › Full Blots/Fig S Full blots Recombinant asay part 2 (2).tif]

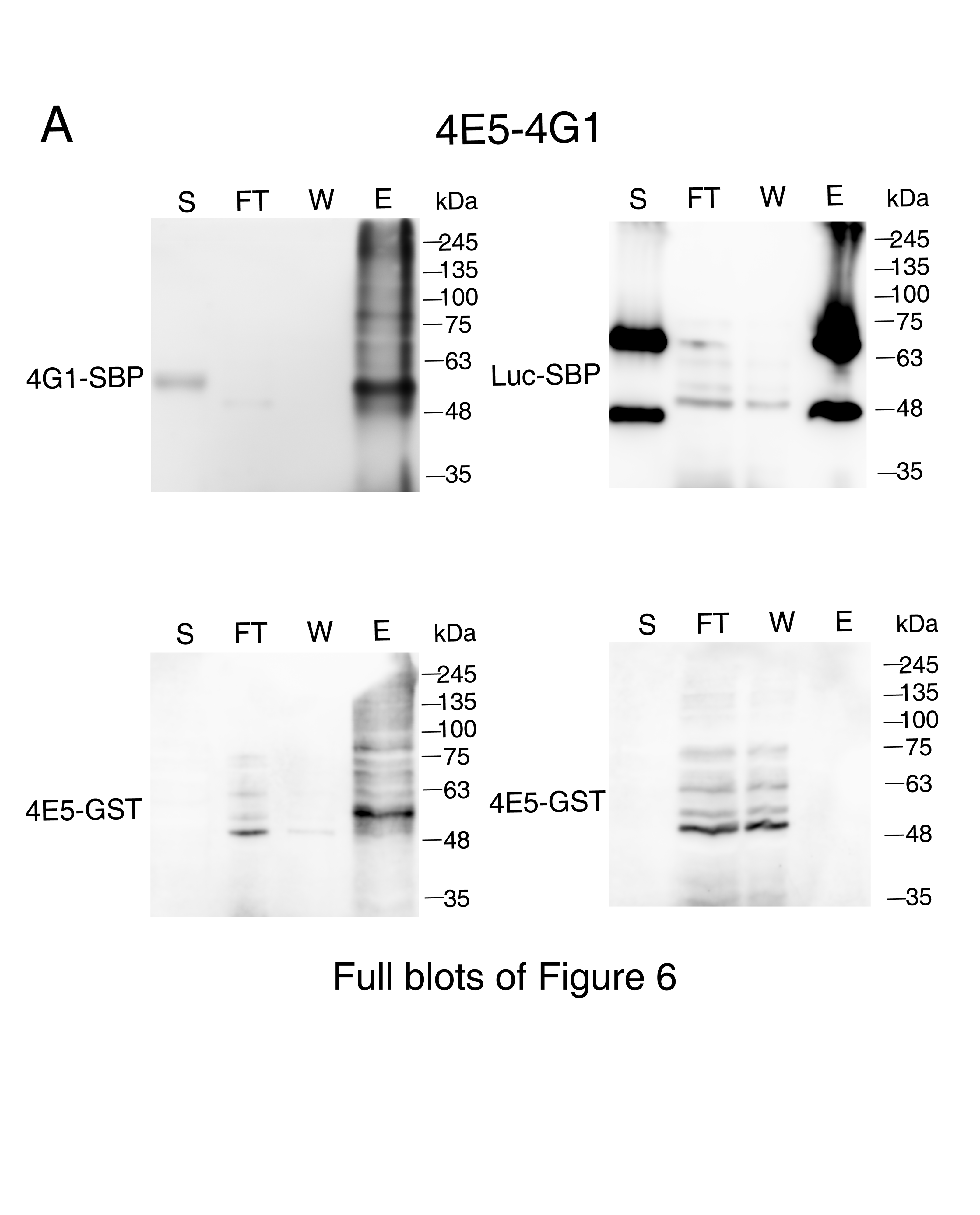

Supplement: Supplementary file 1 [file ijms-22-03979-s001.zip › Full Blots/Fig S Full blots Recombinant asay part 1 (2).tif]

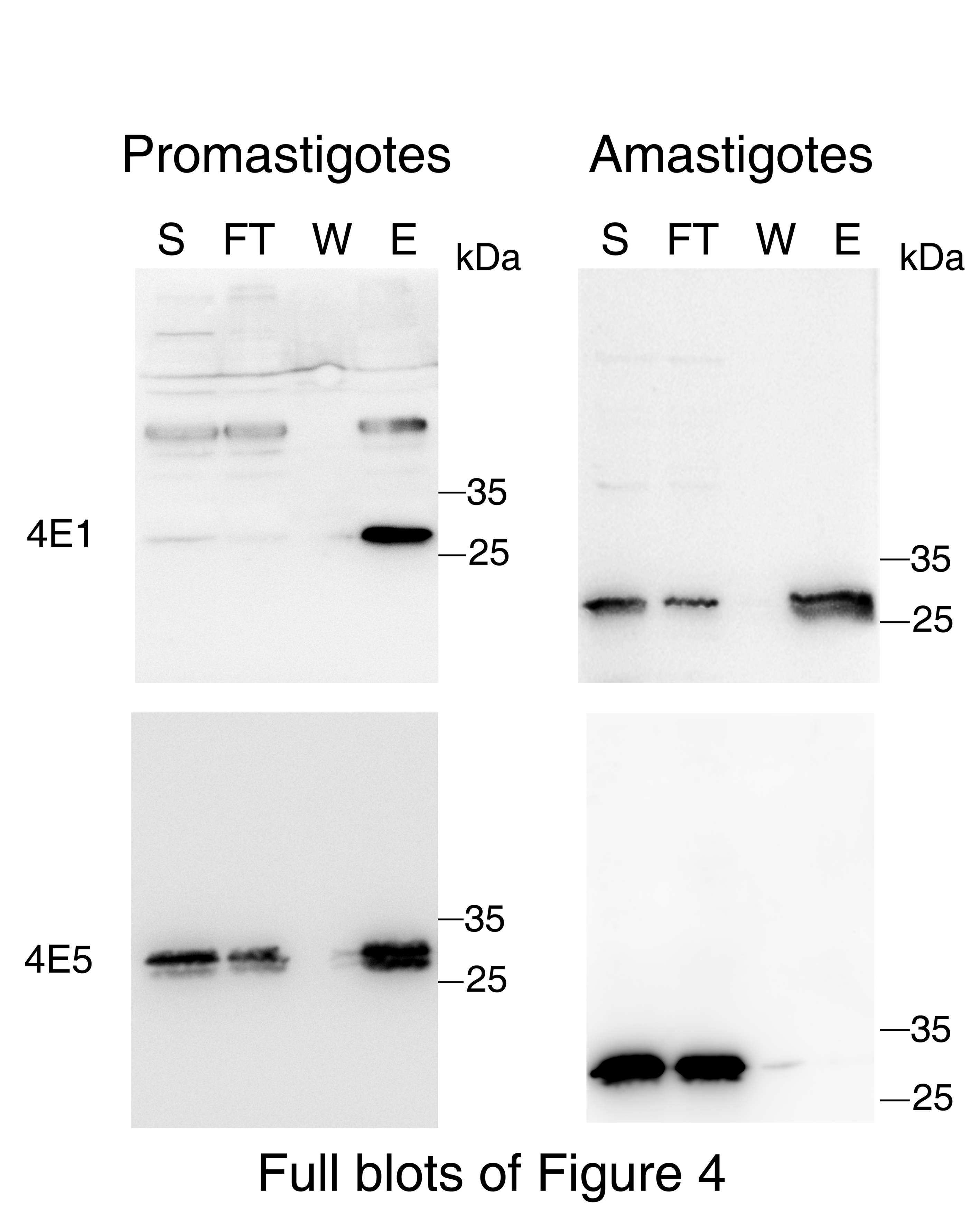

Supplement: Supplementary file 1 [file ijms-22-03979-s001.zip › Full Blots/Fig S Full blots m7GTP assay (1).tif]
